# The SUMOylated RREB1 interacts with KDM1A to induce 5‐fluorouracil resistance via upregulating thymidylate synthase and activating DNA damage response pathway in colorectal cancer

**DOI:** 10.1002/mco2.70105

**Published:** 2025-02-21

**Authors:** Ya‐Nan Deng, Lan Huang, Shan Gao, Zenghua Sheng, Yinheng Luo, Nan Zhang, Samina Ejaz Syed, Ruiwu Dai, Qiu Li, Xianghui Fu, Shufang Liang

**Affiliations:** ^1^ Department of Biotherapy, Cancer Center and State Key Laboratory of Biotherapy, West China Hospital Sichuan University Chengdu PR China; ^2^ Department of Medical Oncology Cancer Center West China Hospital Sichuan University Chengdu PR China; ^3^ Department of Biochemistry and Biotechnology, Baghdad Campus The Islamia University of Bahawalpur Bahawalpur Pakistan; ^4^ Department of General Surgery General Hospital of Western Theater Command Chengdu PR China

**Keywords:** 5‐fluorouracil, chemoresistance, colorectal cancer, DNA damage response, KDM1A, RREB1, SUMOylation

## Abstract

Chemoresistance is one main cause of failure in colorectal cancer (CRC) treatment. The role of transcription factor Ras‐responsive element binding protein 1 (RREB1) remains unclarified in CRC chemoresistance. Herein, we reveal that RREB1 functions as an oncogene to promote cell proliferation and 5‐fluorouracil (5‐FU) chemoresistance in CRC, and SUMOylation is required for RREB1 to exert its oncogenic role in CRC. RREB1 induced cell cycle arrest at the S‐phase and a decreased apoptosis rate under 5‐FU exposure. Mechanistically, the interaction of RREB1 with lysine demethylase 1A (KDM1A) elevated expression of 5‐FU targeting proteins thymidylate synthase (TS) and thymidine kinase (TK1) to maintain the nucleotide pool balance under 5‐FU treatment, and enhanced activation of Chk1‐mediated DNA damage response (DDR) pathway. The deSUMOylation of RREB1 resulted in a reduced interaction of RREB1 with KDM1A, contributing to a downregulation of TS expression and a less activation of DDR pathway. Moreover, KDM1A knockdown improved the DNA damage and reduced RREB1‐mediated resistance to 5‐FU. These findings provide new insights into RREB1‐mediated chemotherapy responses in CRC and indicate RREB1 is a potential target for overcoming 5‐FU resistance.

## Graphical abstract

5‐Fluorouracil (5‐FU) treatment triggers the Ras‐responsive element binding protein 1 (RREB1) expression and the formation of RREB1 and lysine demethylase 1A (KDM1A) complex which binds to the promoter of thymidylate synthase (*TS*) and thymidine kinase (*TK1*), and be recruited to phosphorylated H2AX foci, contributing to reduced DNA damage and enhanced DNA damage response.

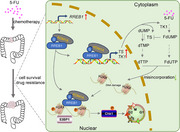

## 1 INTRODUCTION

Colorectal cancer (CRC) ranks as the second leading cause of cancer‐related deaths globally, accounting for 9.4% of fatalities, trailing only lung cancer with 18%. It remains a formidable health challenge, with approximately 1,880,527 new cases diagnosed in 2020.^1^ 5‐Fluorouracil (5‐FU)‐based adjuvant chemotherapy continues to hold sway in CRC treatment. 5‐FU, an antimetabolite, exerts its antitumor activity by disrupting deoxynucleotide (dNTP) pools and incorporating its metabolites into RNA and DNA, thereby triggering DNA damage and cell death.^2^ As one of the most potent DNA‐damaging agents in clinical practice, 5‐FU has significantly bolstered the 5‐year survival rates for CRC patients.^3^ Nevertheless, despite its efficacy and widespread application, 5‐FU faces limitations, including a low response rate among patients and the development of resistance. Consequently, unraveling the mechanisms underlying 5‐FU resistance in CRC adjuvant therapy is imperative to enhancing its therapeutic outcomes.

Moreover, 5‐FU metabolism‐relative genes are also associated with its chemotherapy effectiveness. Thymidylate synthase (TS), which catalyzes the conversion of deoxyuridine monophosphate (dUMP) to deoxythymidine monophosphate (dTMP), exhibits a positive correlation with 5‐FU resistance in CRC. The intermediary, fluorodeoxyuridine monophosphate (FdUMP), forms a stable ternary complex with TS and CH2THF, effectively inhibiting TS activity. Moreover, upregulation of thymidine kinase (TK1) phosphorylase, a pivotal initial gene in converting 5‐FU to fluorodeoxyuridine (FUDR), has been reported to enhance 5‐FU sensitivity.^2^

5‐FU's ability to inflict DNA damage is contingent upon cell cycle and apoptosis, particularly active during the S‐phase, dormant in G0 or G1 phases.^4^ Elevated DNA damage response (DDR) is another mechanism contributing to 5‐FU resistance in CRC, involving pathways including base excision repair, mismatch repair, and homologous recombination.^5^ Incorporation of 5‐FU metabolites into DNA and RNA leads to single‐ and double‐strand breaks, marked by Serine 139‐phosphorylated H2AX (γH2AX), which recruits repair proteins to initiate the DNA repair process.^6^ Modulation of DDR pathway components can sensitize CRC to 5‐FU, thus DDR is a promising therapeutic target in CRC.^7^, ^8^

The multifaceted function of Ras‐responsive element binding protein 1 (RREB1) in cancer progression and metabolic disorders has garnered attention from various research groups. Our prior review outlines RREB1's role in fostering cell growth in pancreatic, prostate, and CRC by modulating target gene expression.^9^ Recent discoveries underscore RREB1's significance in development and fibrosis processes, linking Ras and transforming growth factor beta (TGF‐β) signaling pathways.^10^, ^11^ RREB1's involvement in DDR has also been noted, with RREB1 activating the DDR pathway by binding to the P53 promoter.^12^ Nevertheless, the precise mechanisms underlying RREB1's response to genotoxic stress remain elusive.

Building upon our prior work, which highlighted post‐translational modifications of RREB1 in cancer progression,^13^ herein we identified RREB1 as a SUMOylation substrate related to CRC progression and chemotherapy. In this study, we explored the role of RREB1 and its SUMOylation in CRC progression and chemoresistance to 5‐FU. Mechanistically, RREB1 fosters CRC cell resistance to 5‐FU therapy by targeting TS and TK1, and enhancing DDR pathways by recruiting lysine demethylase 1A (KDM1A) interaction. Our findings provide novel perspectives on RREB1‐mediated chemoresistance in CRC, positioning RREB1 as a potential therapeutic target for overcoming 5‐FU resistance.

## 2 RESULTS

### 2.1 RREB1 is SUMOylated by SUMO1 and SUMO3 conjugation

**FIGURE 1:**
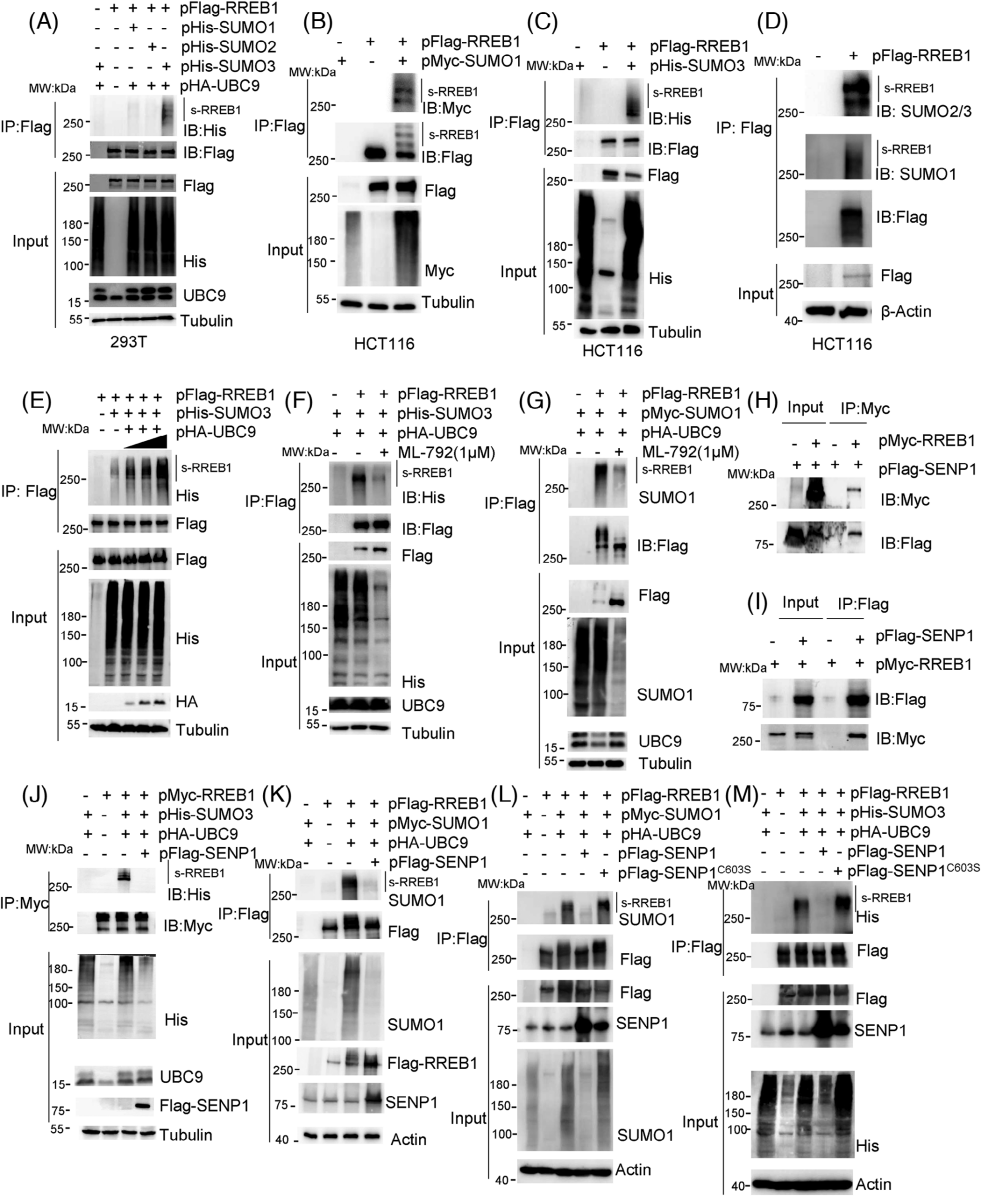
Ras‐responsive element binding protein 1 (RREB1) was SUMOylated by SUMO1 and SUMO3 conjugation in colorectal cancer (CRC) cells. (A) SUMOylation of RREB1 was determined by western blot in HEK293T cells transfected with pFlag‐RREB1 plasmid combined with pHis‐SUMO1, pHis‐SUMO2, or pHis‐SUMO3. (B, C) RREB1 SUMOylation was detected in HCT116 cells. (D) RREB1 modified by endogenous SUMO1 or SUMO2/3 was detected in HCT116 cells stably expressing Flag‐RREB1. (E) RREB1 SUMOylation modified by His‐SUMO3 was increased by pHA‐UBC9 inducement in HCT116. (F, G) A SUMOylation inhibitor ML‐792 inhibited the His‐SUMO3‐modified RREB1 (F) or Myc‐SUMO1‐modified RREB1 (G) of HCT116 cells that were cotransfected with pFlag‐RREB1, pHA‐UBC9, and pHis‐SUMO3/pMyc‐SUMO1 plasmids. (H, I) RREB1 interacted with sentrin‐specific protease 1 (SENP1) in HCT116 cells. pMyc‐RREB1 and pFlag‐SENP1 were cotransfected into HCT116 cells as indicated for 48 h, and co‐immunoprecipitation (Co‐IP) was performed by anti‐Myc antibody (H) or anti‐Flag M2 antibody (I). (J) Flag‐SENP1 overexpression obviously reduced the RREB1 SUMOylation modified by His‐SUMO3 in HCT116 cells. (K) pFlag‐SENP1, pFlag‐RREB1, pHA‐UBC9, and pMyc‐SUMO1 were cotransfected in HCT116 cells for 48 h, then the SUMO1‐modified RREB1 was detected after IP. (L, M) Flag‐SENP1^C60S^ mutant abolished the catalytic analysis of wild‐type Flag‐SENP1 to inhibit the Myc‐SUMO1 (L) or His‐SUMO3 (M) conjugation with RREB1.

Protein SUMOylation assay was conducted in HEK293T cells cotransfected with pFlag‐RREB1 and various SUMO/UBC9‐expressing plasmid constructs to investigate SUMOylation‐mediated regulation of RREB1. As results, RREB1 was indeed a substrate of SUMOylation, primarily conjugated by SUMO3 (Figure 1A), with a minor contribution from SUMO1 conjugation. This result was validated in HCT116 cells, in which RREB1 was SUMOylated by both SUMO1 (Figure 1B) and SUMO3 (Figure 1C). To further explore RREB1 SUMOylation, western blot analysis was performed using antibodies against SUMO1 or SUMO2/3 following immunoprecipitation (IP) with an anti‐Flag antibody in HCT116 cells stably expressing Flag‐RREB1. The results demonstrated that RREB1 was predominantly SUMOylated by endogenous SUMO2/3 in CRC cells (Figure 1D). We have also confirmed that cell endogenous RREB1 undergoes SUMOylation modification in HCT116 cells (Figure S1A). Moreover, the increase of the SUMO conjugating enzyme UBC9 led to an enhancement of the SUMOylated RREB1 protein bands (Figure 1E). In contrast, treatment with ML‐792, a specific inhibitor of SUMO‐activating enzyme 1 (SAE1), led to a reduction of SUMO3‐modified RREB1 levels (Figure 1F) and attenuated SUMO1 conjugation (Figure 1G).

Co‐immunoprecipitation (Co‐IP) experiments were conducted to identify the enzymes in SUMO/sentrin‐specific protease (SENP) family,^14^ that is responsible for RREB1 deSUMOylation. Our results indicated that RREB1 specifically interacted with SENP1 (Figure 1H,I), but not with SENP2 and SENP3 (Figure S1B), suggesting SENP1‐mediated RREB1 deSUMOylation. To validate this, pFlag‐SENP1 was transfected into HCT116 cells. SENP1 overexpression reduced SUMO3‐modified RREB1 levels (Figure 1J), with a similar effect observed for SUMO1‐modified RREB1 (Figure 1K).

Given SENP1 catalytic activity is dependent on cysteine 603,^15^ we generated a noncatalytic SENP1 variant, Flag‐SENP1^C603S^, replacing C603 with serine. Our findings revealed that Flag‐SENP1^C603S^ failed to diminish SUMO1 or SUMO3‐modified RREB1 in HCT116 cells (Figure 1L,M), confirming SENP1‐mediated RREB1 deSUMOylation in CRC cells. In conclusion, our study demonstrates that RREB1 serves as a substrate for SENP1‐mediated deconjugation with SUMO1 and SUMO3 at Lys residues in CRC.

### 2.2 RREB1 is mainly SUMOylated at residues of K551, K885, and K913

**FIGURE 2:**
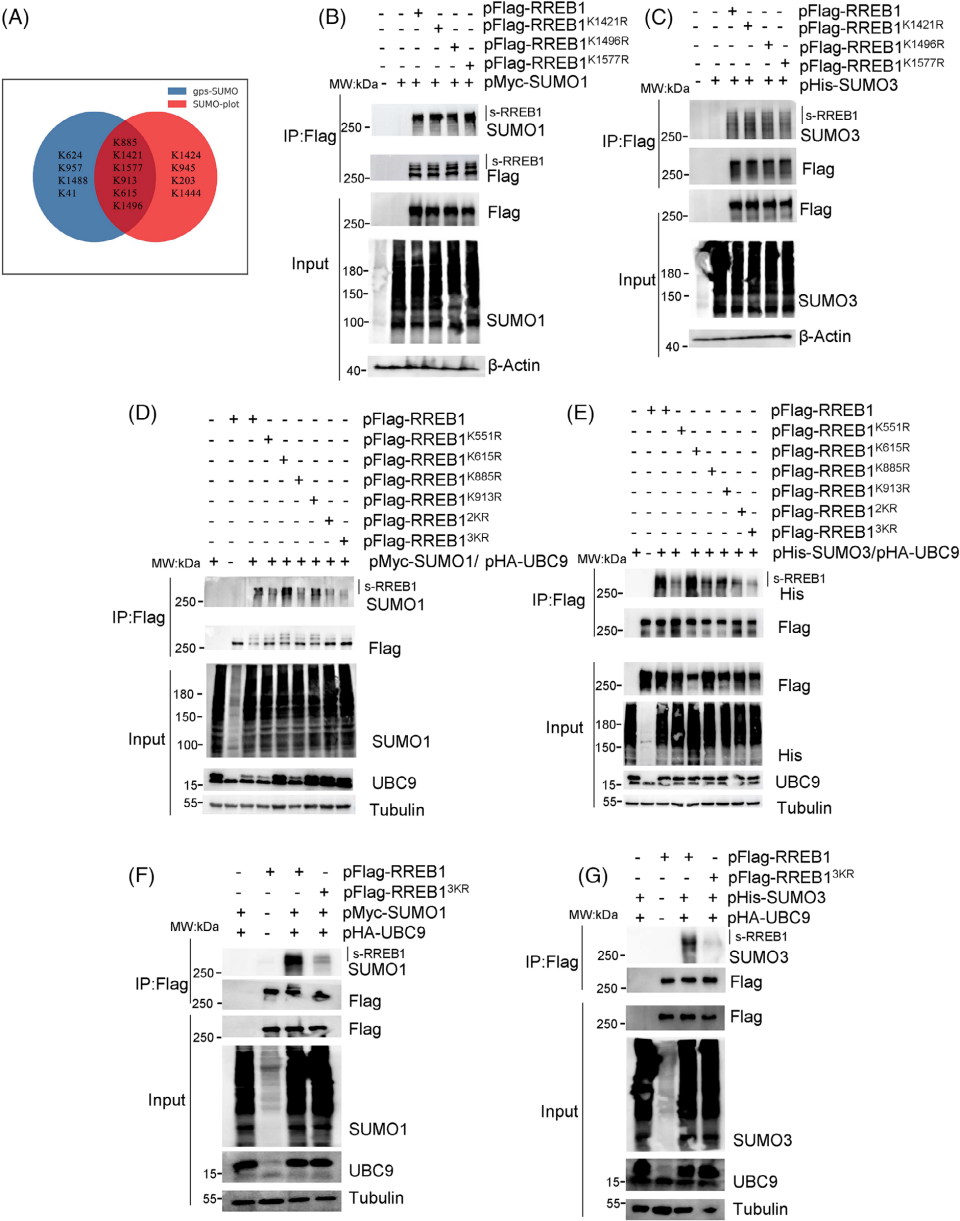
Ras‐responsive element binding protein 1 (RREB1) was mainly SUMOylated at K551, K885, and K913 sites. (A) Prediction of RREB1 SUMOylation using online software SUMOplot (http://www.abgent.com/sumoplot) and gps‐SUMO (http://sumosp.biocuckoo.org/). (B, C) SUMOylation sites were determined by western blot in HEK293T cells. The SUMOylated Flag‐RREB1 or mutant was detected by anti‐SUMO1 (B) or anti‐SUMO2/3 (C) antibody. (D, E) The SUMOylation sites of RREB1 were measured at K551, K615, K885, K913 and their combination in HEK293T cells. Simultaneous mutation of K551 and K885 into arginine to generate Flag‐RREB1^2KR^ or mutations of K551, K885, and K913 into arginine to generate Flag‐RREB1^3KR^. Similarly with panels B, C, pFlag‐RREB1 and mutants were cotransfected with pMyc‐SUMO1 or pHis‐SUMO3 into HEK293T cells for 48 h. (F, G) SUMO1 or SUMO3 modification on Flag‐RREB1 (F) or Flag‐RREB1^3KR^ (G) were detected in HEK‐293T cells.

Two bioinformatics tools, SUMOplot and GPS‐SUMO, were utilized to predict potential SUMOylation sites in RREB1 (Table S1). Six common sites, including Lys‐615, Lys‐885, Lys‐913, Lys‐1421, Lys‐1496, and Lys‐1577, were predicted by the two tools (Figure 2A). In addition, Lys‐551, previously reported as a SUMOylation site by mass spectrometry (MS),^16^ was also included. Sequence alignment revealed conservation of K551, K615, K885, and K913 across species (Figure S2A), hinting at their functional significance.

To verify SUMOylation sites, we generated seven pFlag‐RREB1 mutants, with a K‐to‐R mutation at K551, K615, K885, K913, K1421, K1496, or K1577. Western blot analysis showed minimal differences in SUMO1 (Figure 2B) or SUMO3 (Figure 2C) modification between wild‐type RREB1 and mutants at K1421, K1496, and K1577, suggesting K1421, K1496, and K1577 are not major SUMOylation sites. Obvious reduction of SUMOylated bands of RREB1 was observed in SUMO1 (Figure 2D) and SUMO3 (Figure 2E) modification at RREB1^K551R^ and RREB1^K885R^, while RREB1^K615R^ showed no change and RREB1^K913R^ exhibited a slight decrease. Notably, no single mutation completely abolished RREB1 SUMOylation, indicating potential redundancy among sites.

A double mutant was constructed by mutating K551 and K885 to R (pFlag‐RREB1^2KR^), and a triple mutant was obtained by simultaneously mutating K551, K885, and K913 to R (pFlag‐RREB1^3KR^). As anticipated, the SUMOylation bands of RREB1 in the RREB1^3KR^ mutant were significantly diminished (Figure 2F,G), indicating that K551, K885, and K913 are the primary SUMOylation sites in RREB1.

### 2.3 SUMOylation is required for RREB1 to promote CRC cell proliferation in vitro and in vivo

**FIGURE 3:**
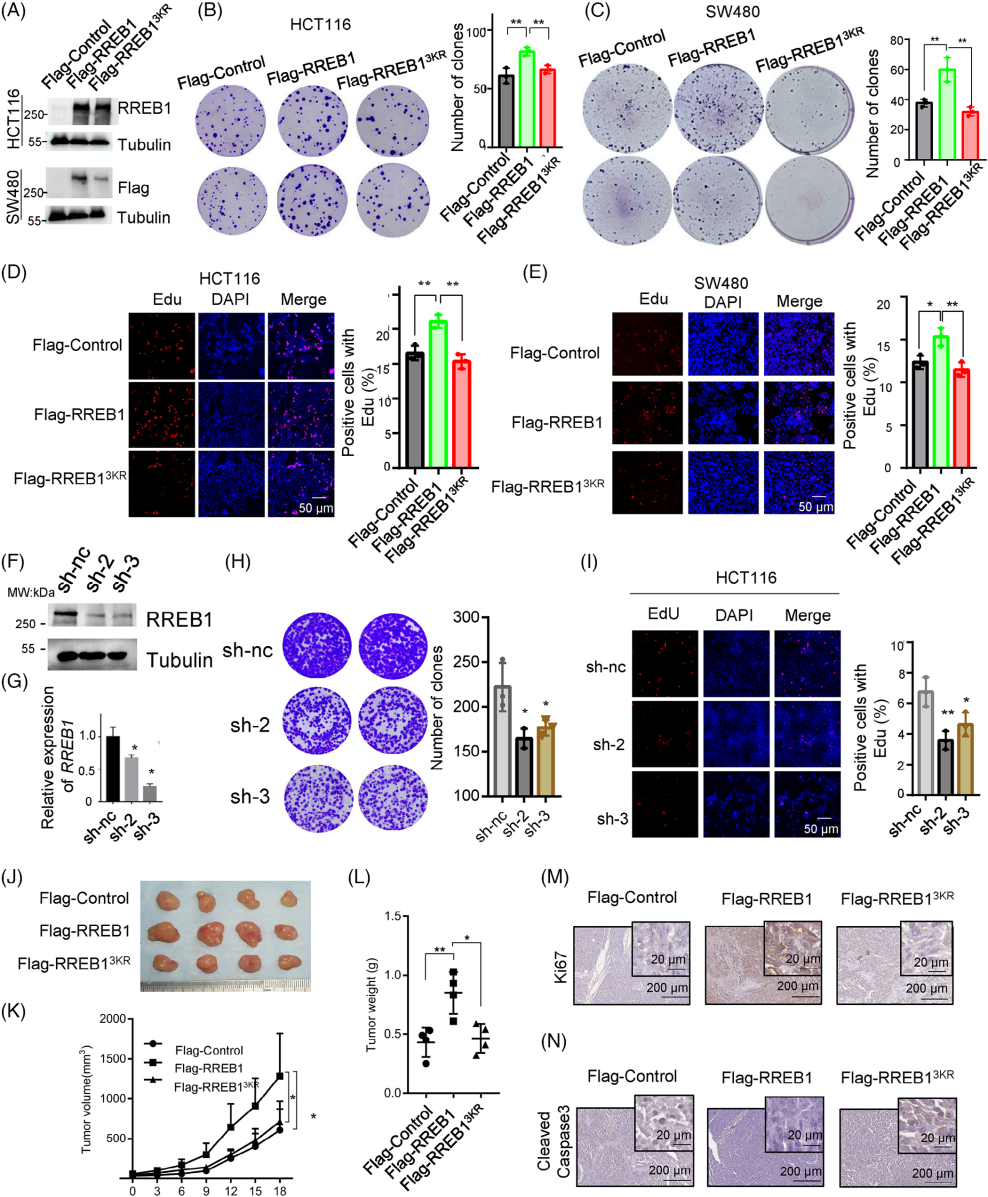
Ras‐responsive element binding protein 1 (RREB1) promotes colorectal cancer (CRC) cell proliferation but RREB1 deSUMOylation retards the effect. (A) Expression of exogenous Flag‐RREB1 and Flag‐RREB1^3KR^ was measured in HCT116 and SW480 cells. Tubulin was used as an internal control. (B, C) A total of 1.0 × 10^3^ cells were seeded in 6‐well plate for 14 days to perform colony formation assay in HCT116 (B) or SW480 (C) cells with overexpressing Flag‐RREB1 or deSUMOylation mutant Flag‐RREB1^3KR^, and with a mock Flag‐Control for negative control, number of clone survivals were counted. (D, E) Cell proliferation was measured by EdU assay. The positive cells with EdU incorporation in HCT116 (D) or SW480 (E) cells with overexpressing Flag‐RREB1 was significantly increased compared with Flag‐control and Flag‐RREB1^3KR^. Scale bars: 50 µm. (F, G) RREB‐knockdown efficiency was measured in HCT116 cells at protein and mRNA levels, respectively. A scramble siRNA sequence (sh‐nc) was as a negative control. (H) 2.0 × 10^3^ cells were seeded in 6‐well plate for 14 days to perform colony formation assay in RREB1‐knockdown HCT116 cells (sh‐2 and sh‐3) and control HCT116 cells (sh‐nc). (I) EdU assay was conducted in RREB1‐knockdown HCT116 cells (sh‐2 and sh‐3) and mock HCT116 cells (sh‐nc) with the same conditions with panel D and E. Scale bars: 50 µm. (J) Xenograft models in BALB/c nude mice bearing Flag‐Control, Flag‐RREB1, or Flag‐RREB1^3KR^‐expressing HCT116 cells. Tumors were isolated and photographed. (K) Tumor volumes were measured after 4–5 days from cell injection, and were recorded every 3 days. (L) Tumor weights were measured after mice euthanized. (M, N) Tumor xenografts in panel J were evaluated for the proliferation marker Ki67 (M) and cleaved‐Caspase3 (N) by immunohistochemistry (IHC). Scale bars: 200 µm and 40× amplification of 20 µm. *p < 0.05, **p < 0.01.

HCT116 and SW480 cells stably overexpressing Flag‐RREB1, Flag‐RREB1^3KR^, or a mock Flag‐Control were established (Figure 3A) to assess the role of RREB1 SUMOylation in CRC progression. Colony formation assays (Figure 3B,C) and EdU assays (Figure 3D,E) revealed that RREB1 overexpression promoted cell proliferation in both cell lines, while RREB1^3KR^ overexpression could not promote CRC proliferation, suggesting RREB1's oncogenic role in CRC is abolished by deSUMOylation. Next, we generated RREB1‐knockdown HCT116 cells using shRNA plasmids (sh‐2, sh‐3) for further investigation (Figure 3F,G). Knockdown of RREB1 significantly reduced clonogenicity (Figure 3H) and EdU‐positive cell proportions (Figure 3I) compared to controls (sh‐nc), reinforcing the oncogenic role of RREB1 SUMOylation in CRC.

We inoculated HCT116 cells stably overexpressing Flag‐RREB1, Flag‐RREB1^3KR^, or Flag‐Control into nude mice to evaluate the effect of deSUMOylation mutant RREB1^3KR^ on tumor growth in vivo. After 18 days, the average tumor volumes of the RREB1 group significantly exceeded those of the Control group (1309 ± 249.3 mm^3^ vs. 608.4 ± 132.3 mm^3^, *p* < 0.05, *n* = 4), whereas the RREB1^3KR^ group remained comparable to Control (641.85 ± 85.54 mm^3^; Figure 3J,K). Postmortem tumor weight measurements concurred, with the RREB1 group exhibiting significantly heavier tumors (0.85 ± 0.08g) than RREB1^3KR^ (0.465 ± 0.11g) and Control (0.43 ± 0.13g; Figure 3L). In line with tumor volumes and weights, RREB1 overexpression elevated Ki67 scores compared to Control and RREB1^3KR^ (Figure 3M). Conversely, the apoptosis marker cleaved‐Caspase 3 was downregulated in RREB1 tumors and upregulated in RREB1^3KR^ tumors (Figure 3N). These findings underscore the oncogenic role of RREB1 in CRC, which is abrogated by deSUMOylation. In summary, our study reveals that RREB1 functions as an oncogene to promote CRC proliferation, and SUMOylation is crucial for this oncogenic activity.

### 2.4 RREB1 improves 5‐FU resistance and RREB1 deSUMOylation sensitizes CRC cell response to 5‐FU‐mediated chemotherapy in vitro and in vivo

**FIGURE 4:**
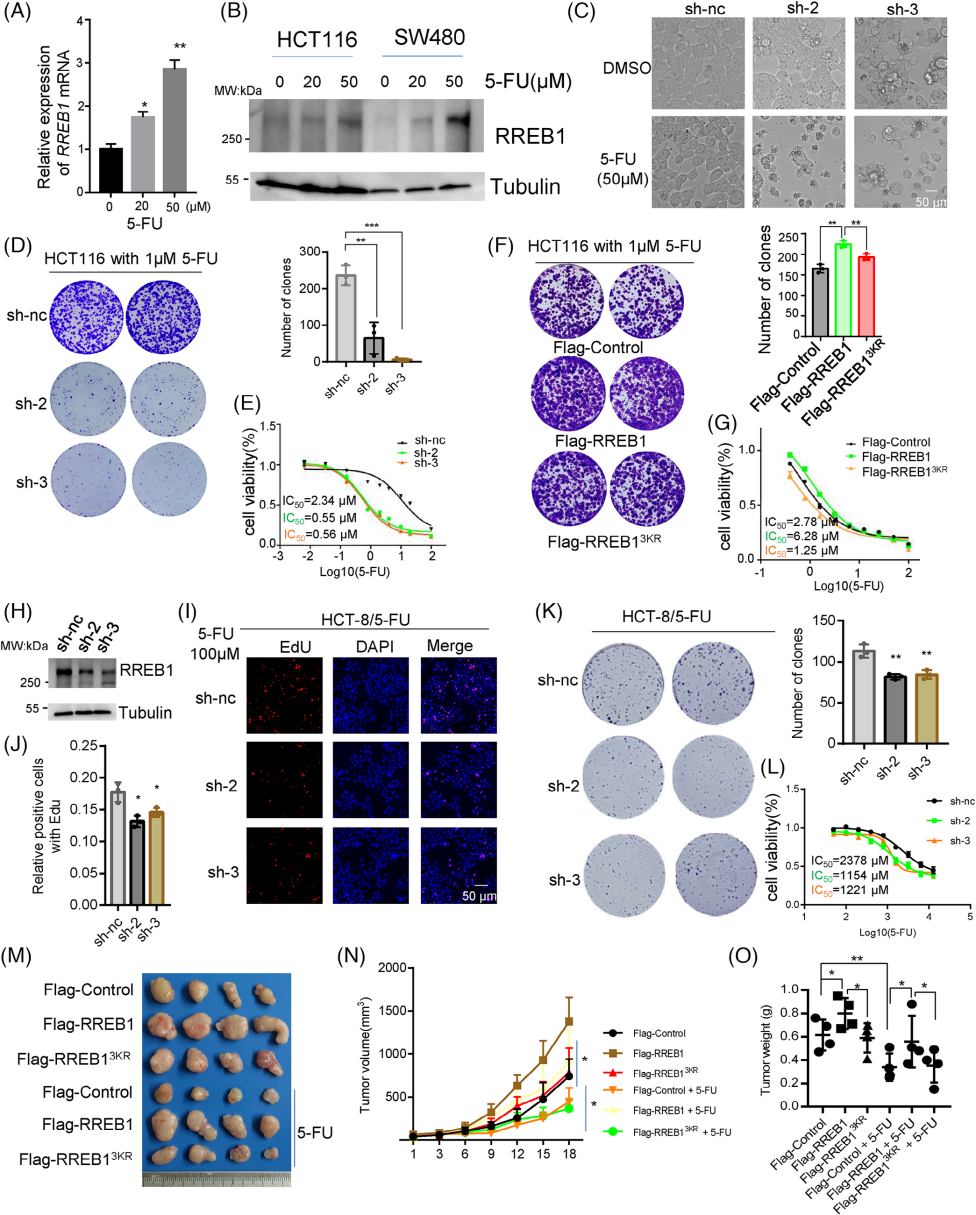
Ras‐responsive element binding protein 1 (RREB1) overexpression contributes to resistance to 5‐fluorouracil (5‐FU) chemotherapy. (A) *RREB1* mRNA detection by (qPCR) in HCT116 cells treated with 0 µM, 20 µM, and 50 µM 5‐FU for 24 h, *β‐actin* was used as an internal control. (B) RREB1 expression at protein level was detected in HCT116 and SW480 cells treated with 5‐FU. (C) Morphological observation of HCT116 cells with RREB1 knockdown (sh‐2 and sh‐3) or with negative control (sh‐nc) after treatment with 50 µM 5‐FU or dimethylsulfoxide (DMSO) for 24 h. Scale bars: 50 µm. (D) Colony formation assay under 1 µM 5‐FU treatment for 14 days in HCT116 cells with RREB1 knockdown (sh‐2 and sh‐3) or with negative control (sh‐nc). (E) RREB1 knockdown in HCT116 cells decreased the IC_50_ of 5‐FU. (F) Colony formation assay under 1 µM 5‐FU treatment for 14 days in HCT116 cells with overexpressing Flag‐RREB1 or Flag‐RREB1^3KR^, as well with Flag‐Control. (G) Cell viability of HCT116 cells was measured to calculate the 5‐FU IC_50_ under treatment of different concentrations of 5‐FU for 48 h. (H) Knockdown efficiency of RREB1 in a 5‐FU‐resistant cell line HCT‐8/5‐FU. (I, J) Cell proliferation was measured by EdU assay under 100 µM 5‐FU treatment in RREB1 knockdown (sh‐2 and sh‐3) and control (sh‐nc) HCT‐8/5‐FU cell lines. Scale bars: 50 µm. (K) Colony formation assay under 100 µM 5‐FU treatment for 16 days in HCT116 cells with RREB1 knockdown (sh‐2 and sh‐3) or with negative control (sh‐nc). (L) 5‐FU IC_50_ detection in RREB1 knockdown (sh‐2 and sh‐3) and control (sh‐nc) HCT‐8/5‐FU cell lines. (M) A total of 2 × 10^6^ cells of HCT116 stably overexpressing Flag‐RREB1, Flag‐RREB1^3KR^, or a Flag‐Control for negative control were subcutaneously injected into nude mice, and then 5‐FU (30 mg/kg) was treated intraperitoneally every 3 days, DMSO was used as a control. (N, O) Tumor volume was measured every 3 days from the day of 5‐FU treatment, and tumor weight was measured after mice euthanized. *p < 0.05, **p < 0.01, ***p = 0.001.

Considering the pivotal role of RREB1 and its SUMOylation in CRC progression, we investigate their influence on CRC cell sensitivity to chemotherapy. First, we assessed mRNA and protein levels of RREB1 in HCT116 and SW480 cells treated with varying concentrations of 5‐FU (0, 20, 50 µM). RREB1 expression was elevated in response to 5‐FU treatment (Figure 4A,B) and oxaliplatin treatment (Figure S3A), implying RREB1 is responsible for chemotherapy sensitivity. RREB1 knockdown in HCT116 cells (sh‐2 and sh‐3) increased 5‐FU‐induced cell death (Figure 4C), significantly decreased the clone formation (*p* < 0.01 and *p* < 0.001, respectively; Figure 4D) and drastically lowered the IC_50_ value for 5‐FU (0.55 ± 0.21 µM and 0.56 ± 0.14 µM for sh‐2 and sh‐3, respectively, vs. 2.34 ± 0.3 µM for sh‐nc; *p* < 0.001 for both; Figure 4E). Similarly, RREB1 knockdown in HCT116 cells reduced survival clones and IC_50_ to oxaliplatin (Figure S3B,C). On the contrary, RREB1 overexpression improved the clone formation numbers and IC_50_ of 5‐FU compared to Flag‐Control (6.28 ± 1.2 µM vs. 2.78 ± 0.89 µM, *p* < 0.05), whereas Flag‐RREB1^3KR^ showed a similar clone survival to Flag‐Control and a lower IC_50_ than Flag‐RREB1 (Figure 4F,G), demonstrating RREB1 is responsible for 5‐FU resistance. The rescue experiment also supports this finding (Figure S3D). A 5‐FU‐resistant cell line HCT‐8/5‐FU was applied to further validate RREB1's role in 5‐FU resistance (Figure 4H). RREB1 knockdown slowed down cell proliferation (Figure 4I,J), decreased the colony formation number (*p* < 0.01 for both sh‐2 and sh‐3; Figure 4K) and IC_50_ to 5‐FU (1154 ± 275.1 µM and 1221 ± 192.2 µM in sh‐2, sh‐3 vs. 2378 ± 475 µM in sh‐nc, *p* = 0.0011 and *p* = 0.001; Figure 4L) or IC_50_ to oxaliplatin (Figure S3E).

RREB1's effect on 5‐FU efficacy was evaluated in nude mice. Thirty mg/kg 5‐FU treatment markedly suppressed tumor growth in Flag‐Control group compared to dimethylsulfoxide (DMSO)‐treated Flag‐Control group (Figure 4M). Within the 5‐FU‐treated group, Flag‐RREB1‐overexpressing HCT116 xenografts exhibited significantly larger tumor volumes and weights than both Flag‐Control and Flag‐RREB1^3KR^‐overexpressing HCT116 xenografts (Figure 4N,O), suggesting RREB1 overexpression promotes 5‐FU resistance. Collectively, these results demonstrated that RREB1 enhances 5‐FU resistance and RREB1 deSUMOylation sensitizes CRC cells to 5‐FU treatment, both in vitro and in vivo.

### 2.5 RREB1 deSUMOylation arrests cell cycle progression and contributes to apoptosis and DNA damage in CRC under 5‐FU treatment

**FIGURE 5:**
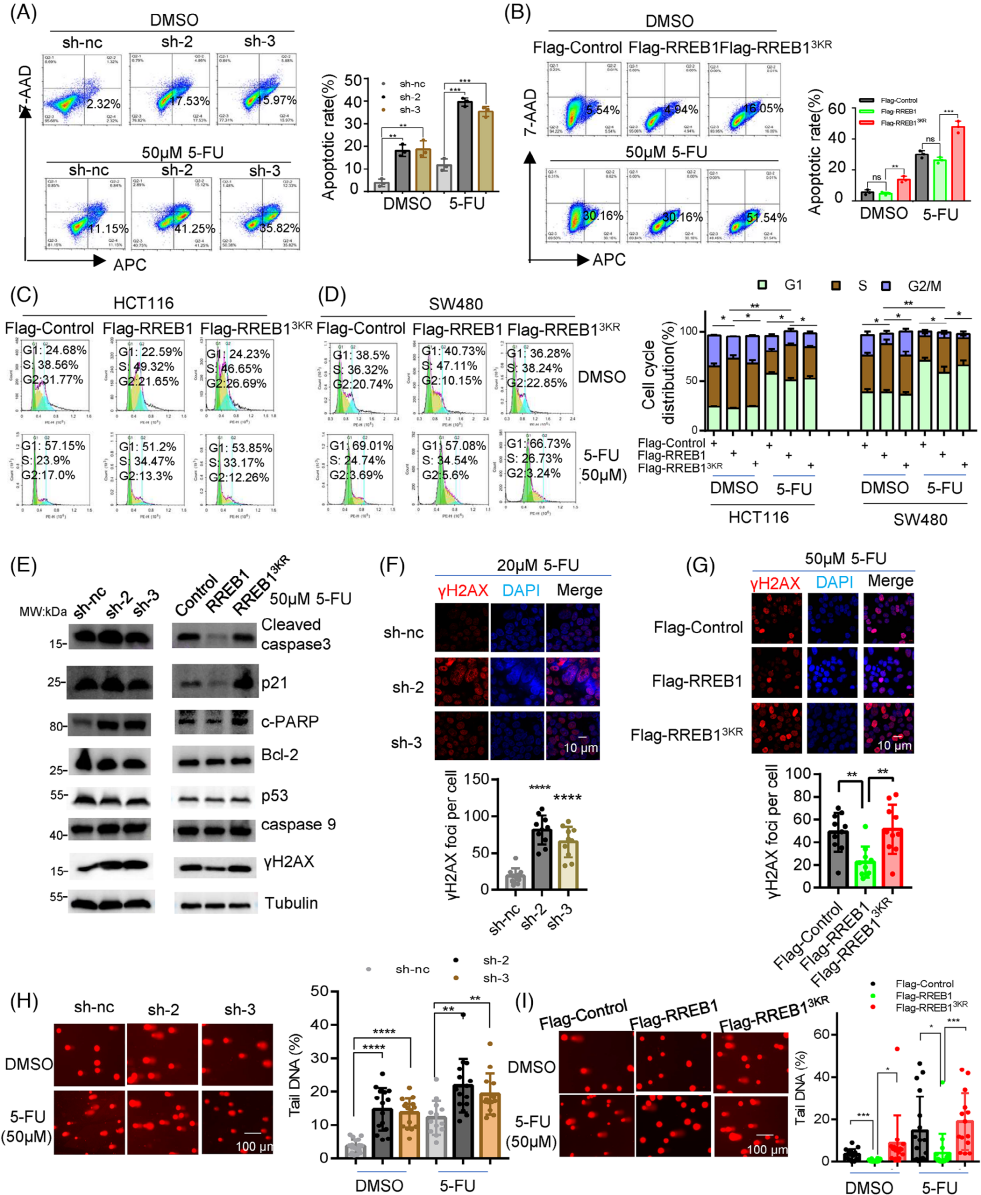
Ras‐responsive element binding protein 1 (RREB1) deSUMOylation arrests cell cycle progression and contributes to increased apoptosis and DNA damage in colorectal cancer (CRC) under 5‐fluorouracil (5‐FU) treatment. (A) Flow cytometry to measure the apoptosis rate in HCT116 cells with RREB1 knockdown (sh‐2 and sh‐3) or with scramble control (sh‐nc) under treatment of 50 µM 5‐FU or dimethylsulfoxide (DMSO) for 24 h. (B) Apoptosis rate was detected in HCT116 cells with overexpressing Flag‐RREB1 or Flag‐RREB1^3KR^ under treatment of 50 µM 5‐FU or DMSO for 24 h. (C, D) Cell cycle detection in HCT116 (C) and SW480 (D) with overexpressing Flag‐RREB1, Flag‐RREB1^3KR^ and with Flag‐Control, with a treatment of DMSO or 50 µM 5‐FU for 24 h. (E) Western blot to detect the expression of proteins involved in apoptosis and cell cycle pathway in RREB1‐knockdown HCT116 cells (sh‐2 and sh‐3) or in HCT116 cells with overexpressing Flag‐RREB1 or Flag‐RREB1^3KR^ after treatment of 5‐FU. (F, G) 5‐FU‐induced DNA damage, marked by γH2AX, was evaluated by immunofluorescence (IF) in RREB1‐knockdown HCT116 cells (F) or in HCT116 cells with overexpressing Flag‐RREB1 or Flag‐RREB1^3KR^ (G). Scale bars: 10 µm. (H, I) Comet assay was conducted to assess the DNA damage in RREB1‐knockdown HCT116 cells (H) or in HCT116 cells with overexpressing Flag‐RREB1 or Flag‐RREB1^3KR^ (I), under treatment with 50 µM 5‐FU or DMSO for 24 h. The tail DNA was measured at least in 10 cells using CASP software. Scale bars: 100 µm. *p < 0.05, **p < 0.01, ***p = 0.001, ****p < 0.0001, ns, not significant.

Previous studies report that 5‐FU triggers cell death by integrating active metabolites into DNA and RNA, ultimately leading to apoptosis and cell cycle arrest.^17^ Consistent with this, our findings revealed a more pronounced apoptotic phenotype in RREB1‐knockdown HCT116 cells treated with 5‐FU (Figure 4C), hinting at RREB1's role in modulating apoptosis. To substantiate this, we conducted a flow cytometry analysis and the result revealed that RREB1 knockdown significantly augmented apoptosis under 5‐FU treatment (Figure 5A). Conversely, RREB1 overexpression tended to diminish apoptosis. Notably, RREB1^3KR^ overexpression markedly enhanced apoptosis in HCT116 cells (Figure 5B). Overexpression of RREB1, but not its mutant RREB1^3KR^, also facilitated HCT116 (Figure 5C) and SW480 (Figure 5D) cell entry into S‐phase. In line with flow cytometry data, 5‐FU treatment significantly upregulated cleaved‐PARP1 in RREB1‐knockdown cells compared to controls, whereas a slight upregulation of cleaved‐caspase 3, p21 and caspase 9. Wild‐type RREB1 overexpression modestly downregulated these markers, whereas RREB1^3KR^ overexpression upregulated them (Figure 5E), revealing that RREB1's deSUMOylation disrupted wild‐type RREB1 ability in CRC cells.

p53, previously identified as an RREB1 target,^12^ did not notably alter its expression in our study, indicating RREB1 operates in a p53‐independent manner here. To investigate RREB1's role in 5‐FU‐induced DNA damage, we applied immunofluorescence (IF) staining on γH2AX, a DNA damage marker,^18^ and comet assay. IF showed that RREB1 knockdown increased the γH2AX foci numbers in HCT116 (Figure 5F) and HCT‐8/5‐FU cells (Figure S4A). Conversely, RREB1 overexpression, but not RREB1^3KR^, decreased the γH2AX foci numbers in HCT116 (Figure 5G) and SW480 (Figure S4B) cells. Under 5‐FU treatment, comet assay showed that RREB1 knockdown improved the tail DNA proportion in HCT116 cells (Figure 5H). RREB1 overexpression, but not RREB1^3KR^_,_ lowered the tail DNA proportion in HCT116 cells (Figure 5I) and SW480 (Figure S4C) cells, suggesting RREB1 safeguards CRC cells from 5‐FU‐mediated DNA damage. The rescue experiment also confirmed this (Figure S4D).

### 2.6 RREB1 promotes expression of TS and TK1 to decrease DNA damage

**FIGURE 6:**
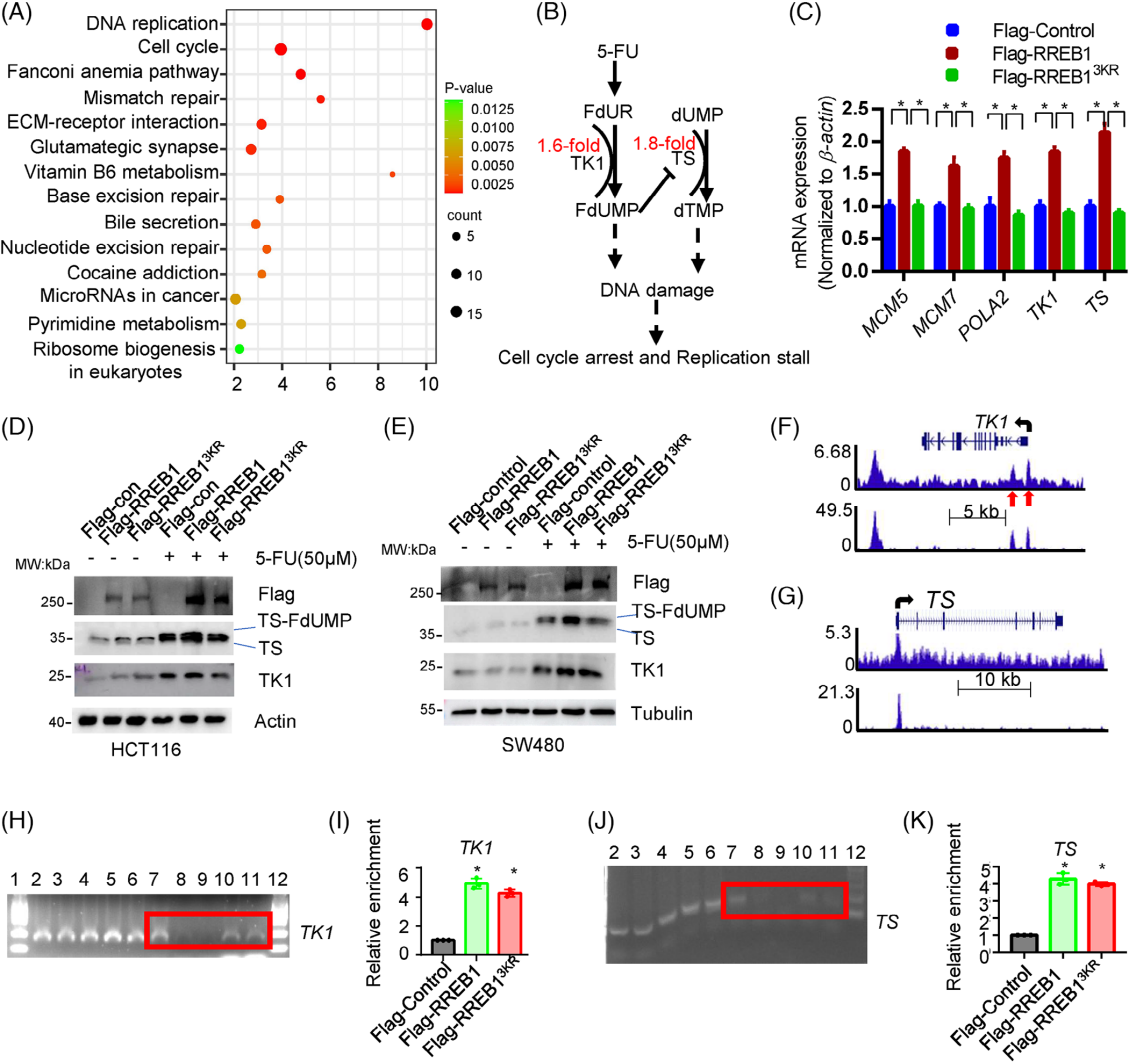
Ras‐responsive element binding protein 1 (RREB1) binds to and promotes the expression of thymidylate synthase (TS) and thymidine kinase (TK1). (A) Kyoto encyclopedia of genes and genomes (KEGG) enrichment of differentially expressed genes (DEGs) identified by transcriptome in Flag‐RREB1 overexpressed HCT116 cells. (B) Diagram of 5‐fluorouracil (5‐FU) metabolism. (C) (qPCR) validation of mRNA expression of *TS*, *TK1*, *POLA2*, *MCM7*, and *MCM5* in HCT116 cells. (D, E) Western blot detection of TK1 and TS expression in RREB‐ or RREB1^3KR^‐overexpression HCT116 (D) or SW480 (E) with or without treatment of 5‐FU. Arrow shows the bands that fluorodeoxyuridine monophosphate (FdUMP) binds irreversibly with TS. (F, G) RREB1 binds to the promoter region of *TK1* (F) and *TS* (G) in K562 cells according to re‐analysis of ChIP‐Seq data in K562 cells (GSE170013 was uploaded to Genome Browser). Red arrows point out the predicted RREB1 binding motif. (H, J) ChIP assay was conducted to confirm the binding of RREB1 on the promoter of *TK1* (H) and *TS* (J) in HCT116 cells. Lane 1: 2000 bp ladder marker; Line 2–4:  input HCT116‐Control; Lane5: input HCT116‐RREB1; Lane6: input HCT116‐RREB1^3KR^; Lane 7: HCT116‐Control positive control (Histone H3 Rabbit mAb); Lane 8: HCT116‐Control negative control (Normal Rabbit mAb); Lane9: HCT116‐Control (Flag‐M2 antibody); Lane10: HCT116‐RREB1 (Flag‐M2 antibody); Lane11: HCT116‐RREB1^3KR^ (Flag‐M2 antibody); Lane12: 2000 bp ladder marker. (I, K) ChIP‐qPCR was performed to detect the enrichment of Flag‐RREB1 or Flag‐RREB1^3KR^ on *TK1* (I) and *TS* (K) promoter region. Data were normalized to the signal of 2.5% input. *p < 0.05.

Transcriptome sequencing was applied to identify RREB1 downstream targets conferring 5‐FU resistance. Kyoto encyclopedia of genes and genomes (KEGG) enrichment analysis highlighted RREB1's impact on cell replication, cell cycle, and various DNA repair pathways, including mismatch, base excision, nucleotide excision repair, and pyrimidine metabolism (Figure 6A), implying its role in cell cycle regulation and DDR. Notably, the upregulation of two genes responsible for the formation of FdUMP and dTMP, TK1 and TS, was validated by quantitative polymerase chain reaction (qPCR; Figure 6B,C). FdUMP, 5‐FU's active metabolite, inhibits TS by irreversible binding, disrupting dUMP‐to‐dTMP conversion and causing nucleotide pool imbalance.^2^ 5‐FU treatment further escalated TK1 and TS expression in HCT116 (Figure 6D) and SW480 (Figure 6E) cells. RREB1 overexpression, but not RREB1^3KR^, improved TS expression under 5‐FU, reducing the disruption of FdUMP on dTMP synthesis, therefore maintaining nucleotide balance and promoting 5‐FU resistance in CRC cells. Conversely, RREB1 knockdown in HCT116 and HCT‐8/5‐FU cells reduced TS and TK1 expression while elevating γH2AX level (Figure S5A,B).

The direct binding of RREB1 to the promoter of *TK1* and *TS* was found in K562 by chromatin immunoprecipitation (ChIP; Figure 6F,G). Subsequently, we validated this in HCT116 cells. Specifically, RREB1 was found to directly bind to *TK1* (Figure 6H,I) and *TS* (Figure 6J,K), validating the findings in K562 cells. Unexpectedly, the enrichment levels of Flag‐RREB1 and Flag‐RREB1^3KR^ in the *TK1* and *TS* promoters were comparable, suggesting that RREB1's deSUMOylation has minimal effect on its DNA binding capability.

### 2.7 RREB1 activates Chk1‐mediated DDR to promote the 5‐FU resistance in CRC

**FIGURE 7:**
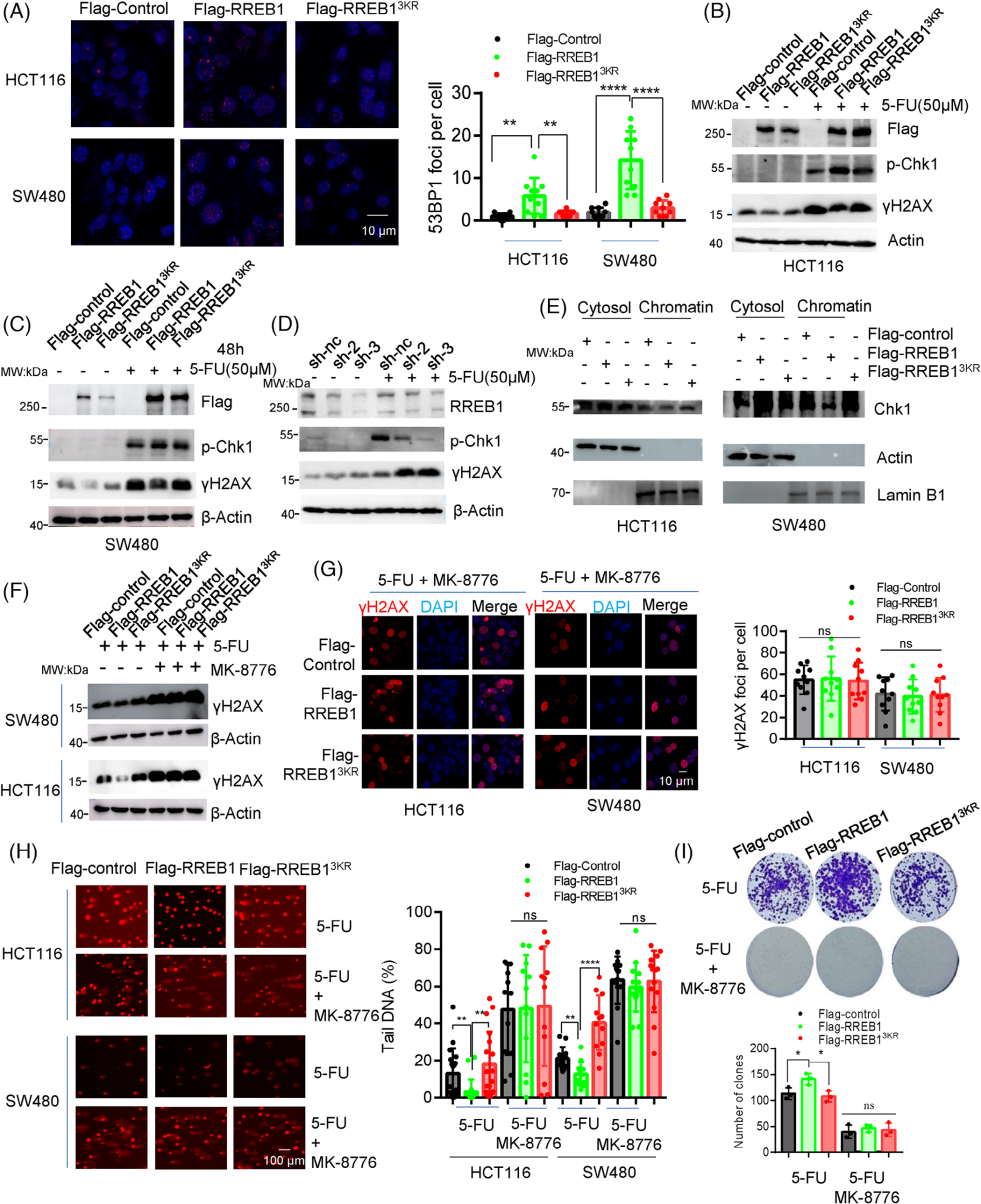
Ras‐responsive element binding protein 1 (RREB1) activates Chk1‐mediated DNA damage response (DDR) to promote the 5‐fluorouracil (5‐FU) resistance in colorectal cancer (CRC). (A) Immunofluorescence (IF) assay to evaluate the effect of RREB1 and RREB1^3KR^ on the recruitment of 53BP1 in HCT116 cells and SW480 cells under treatment of 5‐FU for 24 h. Scale bars: 10 µm. (B–D) RREB1 overexpression or knockdown in HCT116 cells were treated with 50 µM 5‐FU for 24 h. Then, the expression of p‐Chk1 and γH2AX were also detected by western blot. (E) RREB1 accelerates the release of Chk1 from chromatin under 5‐FU treatment. The cytosol and chromatin were isolated using a kit from Beyotime. And the level of Chk1 in cytosol and in chromatin was measured by western blot. (F, G) We treated RREB1‐ or RREB1^3KR^‐overexpressed cells in HCT116 and SW480 with or without 10 µM MK‐8776, a Chk1 inhibitor, in combination with 50 µM 5‐FU for 24 h. Then the level of γH2AX was detected by western blot (F) or IF assay (G). Scale bars: 10 µm. (H) Comet assay was applied to evaluate the effect of Chk1 inhibitor MK‐8776 on 5‐FU‐induced DNA damage. Fifty µM 5‐FU in combination with or without 10 µM MK‐8776 were treated for 24 h. The tail DNA was measured at least in 10 cells using CASP software. Scale bars: 100 µm. (I) Colony formation assay under 1 µM 5‐FU treatment or under 1 µM 5‐FU plus 1 µM MK‐8776 for 14 days in HCT116 cells with overexpressing Flag‐RREB1 or Flag‐RREB1^3KR^. *p < 0.05, **p < 0.01, ****p < 0.0001, ns, not significant.

To evaluate the RREB1‐meidated DDR activation, we performed IF and observed significant 53BP1 foci formation in HCT116 and SW480 cells upon RREB1 overexpression, but not RREB1^3KR^‐overexpression (Figure 7A). Conversely, RREB1 knockdown reduced 53BP1 foci formation (Figure S6A,B). Furthermore, 5‐FU treatment triggers RREB1 to activate phosphorylation of checkpoint 1 (p‐Chk1; Ser345) in HCT116 (Figure 7B) and SW480 (Figure 7C) cells, while inhibiting γH2AX level. In contrast, RREB1‐knockdwon attenuated p‐Chk1 activation and increased γH2AX level (Figure 7D). Consistent with reports that Chk1 activation leads to its release from chromatin to cytosol,^19^ we found that RREB1 overexpression, but not RREB1^3KR,^ reduced chromatin‐bound Chk1 and increased cytosolic Chk1 (Figure 7E). These findings confirm RREB1's role in activating DDR pathway, while the de‐SUMOylation mutant RREB1^3KR^ attenuates this activation.

To confirm that RREB1‐induced 5‐FU resistance stems from Chk1‐mediated DDR activation, we employed the Chk1 inhibitor MK‐8776 to suppress the signal transduction in DDR. Treatment with MK‐8776 abolished the downregulation of γH2AX caused by RREB1 overexpression, as evidenced by western blot (Figure 7F) and IF (Figure 7G). Furthermore, MK‐8776 restored the reduced tail DNA in RREB1‐overexpressed HCT116 and SW480 cells (Figure 7H). In colony formation assays with HCT116 cells overexpressing RREB1 or RREB1^3KR^ under 5‐FU treatment, MK‐8776 reduced clone numbers and negated the increase in clones caused by RREB1 in the absence of the inhibitor (Figure 7I). Collectively, these results demonstrate that RREB1's contribution to 5‐FU resistance in CRC is contingent upon Chk1‐mediated DDR activation.

### 2.8 KDM1A cooperates with SUMOylated RREB1 to promote expression of 5‐FU targets and to activate Chk1‐mediated DDR

**FIGURE 8:**
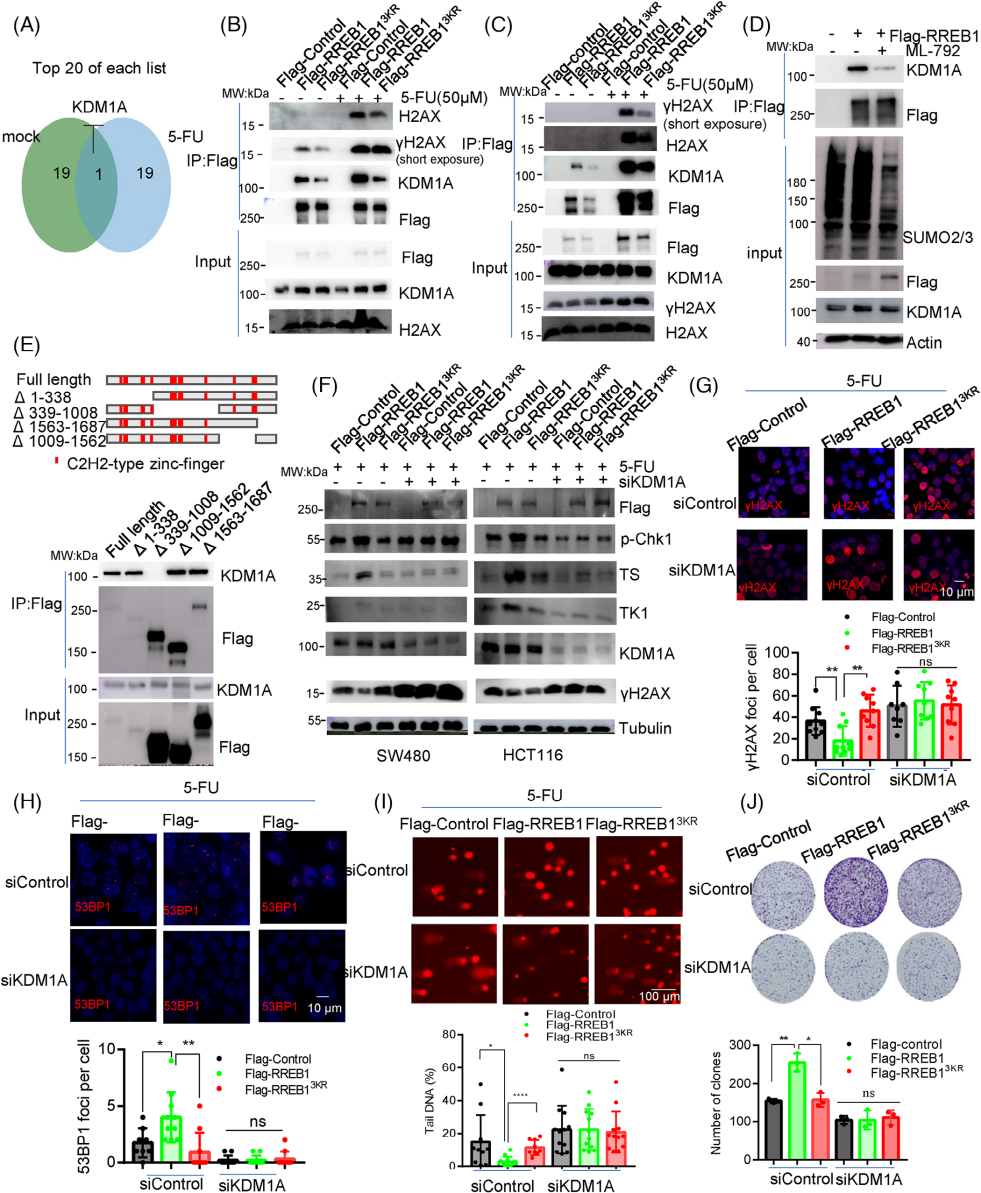
Lysine demethylase 1A (KDM1A) cooperates with SUMOylated Ras‐responsive element binding protein 1 (RREB1) to promote the expression of 5‐fluorouracil (5‐FU) targets and to activate Chk1‐mediated DNA damage response (DDR). (A) Mass spectrometry identification of proteins interacting with RREB1 under 50 µM 5‐FU treatment for 24 h or in normal condition (mock) in HCT116 cells with overexpressing Flag‐RREB1. Co‐immunoprecipitation (Co‐IP) was conducted with anti‐Flag antibody. (B, C) Validation of interaction in HCT116 (B) and HEK293T (C) cells in normal condition and 5‐FU treatment. (D) ML‐792 attenuated the interaction between RREB1 and KDM1A. (E) We generated Flag‐tagged RREB1 truncated mutants to explore the fragment within RREB1 that is required for interaction with KDM1A. (F) We transfected pFlag‐Control, pFlag‐RREB1, or pFlag‐RREB1^3KR^ into KDM1A‐interfering colorectal cancer (CRC) cells. After 48 h, 5‐FU was added to culture at a final concentration at 50 µM for further incubation for 24 h. Then total proteins were collected for western blot analysis. (G, H) Under 5‐FU treatment, effect of RREB1 and RREB1^3KR^ on formation of γH2AX foci (G) or 53BP1 foci (H) were measured by immunofluorescence (IF) assay after KDM1A was interfered. siControl is a scramble sequence control. Scale bars: 10 µm. (I) Comet assay was applied to evaluate the effect of KDM1A silencing on RREB1‐mediated DNA damage protection. Scale bars: 100 µm. (J) Colony formation assay under 1 µM 5‐FU treatment for 10 days. *p < 0.05, **p < 0.01, ****p < 0.0001, ns, not significant.

The deSUMOylation mutant RREB1^3KR^ exhibited comparable affinity to wild‐type RREB1 in binding to target gene promoters (Figure 6E,F), hinting at SUMOylation's potential influence on RREB1's ability to recruit other proteins. To decipher this effect, we employed MS to identify RREB1‐interacting proteins under normal and 5‐FU‐treated conditions, and found that KDM1A ranked prominently in the 5‐FU treatment group (Figure 8A). The interaction between RREB1 and KDM1A was confirmed under normal conditions, and this interaction intensifies upon 5‐FU treatment, concomitant with the appearance of H2AX and γH2AX interactions (Figure 8B,C). Unlike wild‐type RREB1, the deSUMOylation mutant RREB1^3KR^ exhibited weakened interactions with KDM1A and γH2AX, revealing that SUMOylation is crucial for RREB1's capacity to recruit these proteins. Figure 8D reveals that ML‐792 treatment significantly reduced SUMOylated RREB1 levels, leading to decreased KDM1A interaction. IF analysis further corroborated the colocalization of RREB1 and γH2AX (Figure S7A). To clarify whether KDM1A binds directly to γH2AX or via RREB1 as a scaffold, we conducted a Co‐IP using an anti‐γH2AX antibody. Our results demonstrated that Flag‐tagged RREB1 and KDM1A could all be detected in the Co‐IP eluates (Figure S7B), indicating that RREB1 functions as a bridge, facilitating the interaction between KDM1A and γH2AX.

RREB1 truncated mutants assay showed that KDM1A interacted exclusively with all mutants except Δ339–1008 (Figure 8E), confirming that the 339–1008 region is the KDM1A binding site, consistent with the deSUMOylation mutant site in RREB1^3KR^ that disrupts KDM1A recruitment under 5‐FU treatment. Thus, SUMOylation is crucial for RREB1's ability to recruit partner proteins.

To assess KDM1A's role in RREB1‐mediated 5‐FU resistance in CRC, we examined expression of TK1 and TS, p‐Chk1 activation, and DDR responses upon KDM1A silencing in HCT116 and SW480 cells. Figure 8F shows that RREB1 failed to upregulate TK1, TS, and p‐Chk1 activation after KDM1A knockdown, indicating KDM1A's involvement. Moreover, KDM1A silencing abolished RREB1's ability to reduce γH2AX foci formation (Figure 8G) and impaired RREB1‐mediated 53BP1 recruitment to DNA damage sites (Figure 8H), highlighting its significance in RREB1‐induced genomic DNA protection (Figure 8I). Ultimately, KDM1A silencing diminished RREB1‐caused 5‐FU resistance (Figure 8J), confirming that RREB1's acquisition of 5‐FU resistance in CRC is KDM1A‐dependent.

### 2.9 RREB1 upregulation indicates a worse prognosis in CRC

**FIGURE 9:**
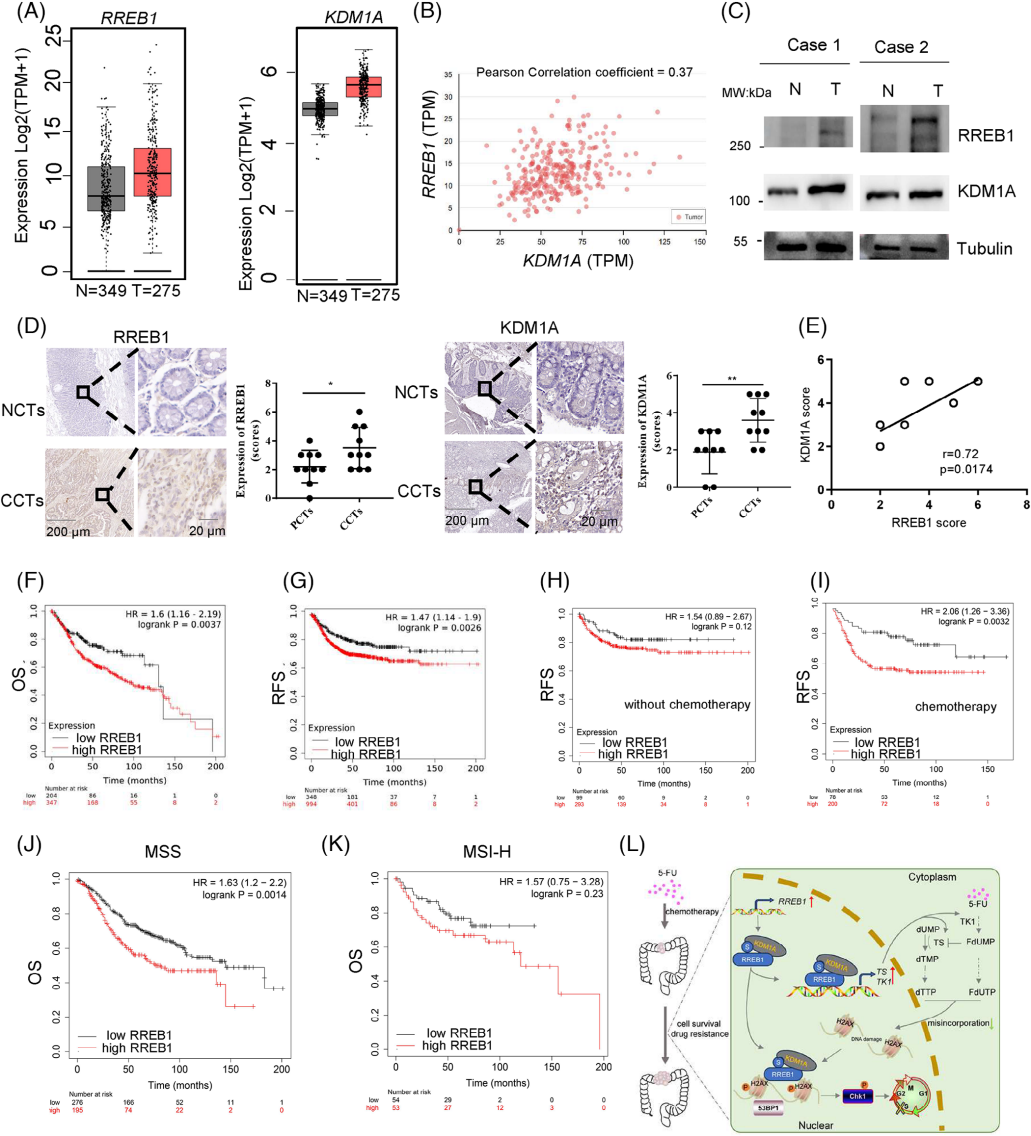
Combination of Ras‐responsive element binding protein 1 (RREB1) and lysine demethylase 1A (KDM1A) predicts a worse prognosis in colorectal cancer (CRC). (A) mRNA expression of RREB1 and KDM1A in CRC using GEPIA (http://gepia.cancer‐pku.cn/). (B) Correlation between *RREB1* and *KDM1A* was analyzed in colon adenocarcinoma dataset (*n* = 286, Pearson correlation coefficient = 0.37) on UALCAN website (http://ualcan.path.uab.edu/). (C) RREB1 and KDM1A protein levels were measured by western blot in two pairs of CRC cases. N, adjacent tissue. T, tumor. (D) Immunohistochemistry (IHC) staining of RREB1 and KDM1A protein in our collected 10 paired colorectal cancer tumors (CCTs) and adjacent noncancerous tissues (NCTs; scale bar, 200 µm and an amplification 20 µm). Right panel shows the statistic of score of each slide. (E) Correlation analysis of RREB1 IHC score and KDM1A IHC score. (F, G) Overall survival (OS) and relapse‐free survival (RFS) analysis in an online website (www.kmplot.com/) based on colon cancer datasets. (H, I) RFS analysis in colon cancer patients with or without adjuvant chemotherapy in online website (www.kmplot.com/). Patients were divided into two groups based on median RREB1 mRNA expression. (J, K) We also performed OS analysis in colon cancer patients based on microsatellite status. MSI, microsatellite instability; MSS, microsatellite stability. (L) A mechanistic schematic diagram of RREB1 in response to 5‐fluorouracil (5‐FU) treatment in CRC (conducted by ScienceSlides PPT plug‐in). Briefly, 5‐FU treatment triggers the RREB1 expression and the complex formation between RREB1 and KDM1A. The complex binds to the promoter of thymidylate synthase (*TS*) and thymidine kinase (*TK1*) and can be recruited to phosphorylated H2AX foci, which contributes to reduced DNA damage and enhanced DNA damage response. *p < 0.05, **p < 0.01, ****p < 0.0001, ns, not significant. SUMOylation phosphorylation.

To gain insights into the prognostic significance of *RREB1* and *KDM1A* in CRC, we analyzed their expression and correlations with clinical outcomes using online databases and our tissue samples. The mRNA expression of both *RREB1* and *KDM1A* was upregulated in CRC patients (T = 275, N = 349; Figure 9A), with a positive correlation between them (Pearson correlation coefficient = 0.37; Figure 9B). At protein level, similar results were achieved in our collected CRC samples through western blot (Figure 9C) and immunohistochemistry (IHC) analysis (Figure 9D), further corroborating their positive correlation (*r* = 0.7263, *p* < 0.05; Figure 9E).

An online survival analysis disclosed that CRC patients with elevated RREB1 expression exhibited shorter overall survival (OS; Figure 9F) and relapse‐free survival (RFS; Figure 9G) in comparison to those with low expression. To assess the predictive potential of RREB1 for adjuvant chemotherapy efficacy, we stratified patients based on treatment receipt and plotted RFS curves. No significant correlation was found between RREB1 expression and RFS among untreated patients (Figure 9H, log‐rank *P* = 0.12). Conversely, in those receiving adjuvant chemotherapy, high RREB1 expression correlated with shorter RFS, while low expression was associated with longer RFS (Figure 9I, log‐rank *P* = 0.0032), suggesting RREB1 as an adverse factor for adjuvant therapy, in line with its 5‐FU tolerance‐enhancing mechanism.

Further, we examined the combined effect of RREB1 and KDM1A on CRC prognosis. Notably, high RREB1 expression predicted shorter OS and RFS irrespective of KDM1A levels (Figure S8A,B), emphasizing RREB1's independent prognostic value. Additionally, utilizing cBioPortal, we identified a frequent RREB1 mutation (10%) in CRC patients, previously reported.^20^ This mutation significantly correlated with increased microsatellite instability‐high (MSI‐H) status (7/11 in mutants vs. 17/95 in wild type, *p* = 0.0024; 17/30 in mutants vs. 74/499 in wild type, *p* < 0.0001; Figure S9A,B). Specifically, RREB1 expression was upregulated in MSI‐type CRC tumors, as evident from TCGA and GSE39582 datasets (Figure S9C).

Next, we delved into the predictive capacity of RREB1 for OS across varying microsatellite statuses in CRC. In microsatellite stability (MSS) CRC, heightened RREB1 expression correlated with reduced OS (Figure 9J). Conversely, in MSI CRC, no significant link was observed between RREB1 and OS (Figure 9K). We hypothesize that the elevated mutation rate of RREB1 in MSI‐high (MSI‐H) tumors may disrupt its normal function, diminishing its oncogenic potential in CRC. This aligns with our prior discovery of RREB1's carcinogenic role in CRC. In conclusion, these findings underscore high RREB1 expression as an adverse prognostic indicator for CRC patient outcomes.

## 3 DISCUSSION

Our study investigates RREB1's role in CRC progression and drug resistance, confirming its oncogenic activity. Survival data show a link between elevated RREB1 and worse OS, RFS in CRC. Importantly, we suggest SUMOylation is crucial for RREB1 function in CRC. Using bioinformatics, conservation analysis, and experiments, we verified SUMO3‐mediated SUMOylation of RREB1 in CRC cells. SUMOylation is implicated in subcellular localization, protein stability, transcription, and DNA repair.^21^, ^22^ While not altering nuclear localization (Figure S10A–C), SUMOylated RREB1 modulates its transcriptional activity, controlling target genes (TS, TK1) by binding their promoters and reducing KDM1A recruitment.

We have elaborated in detail on the mechanism of how RREB1 enhances the resistance of CRC to 5‐FU. Several mechanisms are known to contribute to 5‐FU resistance, including incorporation of 5‐FU metabolites into DNA and RNA, enhanced DNA repair function and reduced 5‐FU uptake.^2^ RREB1 mainly enhances the tolerance of CRC to 5‐FU in two ways. On the one hand, RREB1 directly transcriptionally regulates key enzymes involved in the conversion of 5‐FU, such as TS and TK1, to reduce the impact of 5‐FU on the balance of the nucleotide pool. TS serves as a pivotal target for 5‐FU's anticancer action. 5‐FU's active metabolite, FdUMP, forms a stable covalent bond with TS, hindering its function, disrupting nucleotide balance, and damaging DNA. Extensive studies highlight high TS expression as a significant contributor to 5‐FU resistance.^23^ TK1, essential for 5‐FU's toxicity, converts FUDR to FdUMP, enhancing DNA integration or TS binding for DNA harm. While TK1 overexpression boosts FdUMP production, it paradoxically enhances 5‐FU resistance by activating the dTTP salvage pathway.^24^

RREB1 enhances DDR pathway activation at DNA damage sites, aiding in repair. Though it was thought to inhibit p53 in cancer radiation therapy,^12^ we show RREB1 does not affect p53 expression (Figure 5G), indicating 5‐FU resistance is p53‐independent. RREB1 binds both γH2AX (damaged DNA marker) and unphosphorylated H2AX, suggesting broader binding patterns. 5‐FU treatment boosts RREB1–γH2AX interaction, recruiting KDM1A, a demethylase maintaining open chromatin crucial for chemo/radioresistance.^25^ This also impacts recruitment of DNA repair proteins like 53BP1, activating Chk1 phosphorylation. RREB1‐KDM1A interaction persists under normal conditions^26^ and is linked to Ras‐Erk pathway gene regulation.^27^ Knockdown of KDM1A reduces RREB1 target genes TS and TK1, implying KDM1A recruitment to RREB1‐targeted promoters. Our findings reveal RREB1 promotes 5‐FU resistance in CRC through multifaceted mechanisms (Figure 9L).

Intriguingly, our RREB1 mutation analysis revealed a ∼10% mutation rate in colon cancer, with MSI significantly more prevalent in mutated cases. This trend mirrors findings in gastric cancer, prompting our MSI‐RREB1 linkage hypothesis. TCGA data analysis corroborated this, showing elevated RREB1 expression in MSI colon cancer patients. Given MSI's genomic instability roots in impaired DNA repair, a RREB1‐DNA repair connection emerges.

While elucidating RREB1 and its SUMOylation's roles in 5‐FU response, our study falls short in exploring RREB1‐targeted drug development for CRC. Moreover, 5‐FU's broad target spectrum limits single‐target enhancement, requiring further validation. The deficiency lies in the fact that this study does not clarify the specific type of DNA repair mediated by RREB1, and the role of SENP1 in RREB1‐mediated 5‐FU resistance has not been confirmed. Nevertheless, we have laid groundwork for future research. Notably, RREB1 mutations strongly correlate with MSI in CRC, a pivotal marker influencing 5‐FU and immunotherapy efficacy. Thus, investigating RREB1's immunotherapy response in CRC holds significant promise.

## 4 MATERIALS AND METHODS

### 4.1 Cell culture

HEK293T and CRC cell lines including HCT116, SW480 were cultured in Dulbecco's modified Eagle medium (DMEM) supplemented with 10% fetal bovine serum (FBS), 100 U/mL penicillin and 100 µg/mL streptomycin at 37°C. 5‐FU‐resistant cell line HCT‐8/5‐FU (CAT# NC0002) and its parental HCT‐8 cell line (CAT# CL0127), ordered from Hunan Fenghui Biotechnology Co., Ltd., were cultured in RPMI‐1640 supplemented with 10% FBS and 100 U/mL penicillin and 100 µg/mL streptomycin. Cell STR identification reports for HEK‐293T, HCT116, SW480, and HCT‐8 were provided.

### 4.2 Establishment of stable RREB1 overexpression or knockdown cell lines

Cell lines stably overexpressing Flag‐tagging RREB1(Flag‐RREB1) or Flag‐tagging RREB1^3KR^ (Flag‐RREB1^3KR^), a triple mutant replacing lysine by arginine at site 551, 885, and 913, in HCT116 and SW480 were constructed using a transpose system. Briefly, 2 µg transposase plasmid Super PiggyBac Transposase and 4 µg RREB1 transposon plasmid (pFlag‐RREB1) containing RREB1 cDNA (GI:1519315172) were cotransfected into HCT116 and SW480 cells for 24 h, following under 1 µg/mL puromycin exposure for 1 week.

*RREB1* gene knockdown cell line was generated with lentivirus infection containing shRNA plasmids targeting *RREB1* (sh‐2 and sh‐3) or scrambled sequence (sh‐nc) for control followed by 1 µg/mL puromycin selection.

### 4.3 Western blot

Cell lysates and sodium dodecyl‐sulfate polyacrylamide gel electrophoresis (SDS‐) analysis was referred our previous methods.^13^, ^28^ Specific primary antibodies were included rabbit anti‐RREB1 (A303‐129A, Bethyl), rabbit anti‐SUMO1 (ET1606‐53, HuaBio), rabbit anti‐SUMO2/3 (ET1701‐17, HuaBio), rabbit anti‐UBC9 (ET1610‐21, HuaBio), mouse anti‐Flag (F1804, Sigma‐Aldrich), His (HRP‐66005, Proteintech), rabbit anti‐TK1 (ET1702‐31, HuaBio), rabbit anti‐TS (ET1705‐24, HuaBio), rabbit anti‐KDM1A (R24798, zenbio), rabbit anti‐p‐Chk1 (HA721189, HuaBio), rabbit anti‐H2AX (AF6187, Affinity), and rabbit anti‐γH2AX (ET1602‐2, HuaBio). Mouse anti‐β‐Tubulin (TA‐10, Zsbio) and mouse anti‐β‐Actin (TA‐09, Zsbio) were used for internal control. Secondary antibodies were diluted at 1:10,000.

### 4.4 SUMOylation detection

RREB1 SUMOylation detection was referred previous methods.^29^ Briefly, 1 × 10^7^ HEK293T cells were harvested and lysed in IP buffer (Beyotime) supplemented with a final concentration at 1 × protease inhibitor cocktail (P1005, Beyotime) and 20 mM N‐ethylmaleimide (NEM; HY‐D0843, Sigma). The supernatant of lysate was incubated with magnetic beads for overnight at 4°C to capture target proteins. The complex of anti‐Flag beads (HY‐K0207‐1, MCE) or Anti‐Myc beads (HY‐K0206‐1, MCE) was washed at least 5–8 times with TBST buffer. Eluted proteins were used for further immunoblotting detection.

### 4.5 Quantitative RT‐PCR

Quantitative reverse transcription polymerase chain reaction (RT‐PCR) was performed using ChamQ SYBR qPCR Master Mix (Q311‐02/03, Vazyme) in BIO‐RAD CFX96 Real‐Time system following manufacturer's instructions (Bio‐Rad Laboratories). *β‐Actin* was used as a loading control to calculate the relative expression level of target genes. Primer sequences were listed in Supporting Information file (Table S2).

### 4.6 Immunohistochemistry staining

IHC staining was referred our previous methods.^30^ Specific antibodies for IHC staining include anti‐RREB1 (A303‐129A, Bethyl), anti‐Ki67 (ET1609‐34, HuaBio) and anti‐cleaved‐caspase3 (ET1602‐47, HuaBio). IHC score was calculated by staining intensity and extent of staining. Staining intensity was defined as following: 0 (negative), 1 (weak), 2 (medium), 3 (strong). The extent of staining was scored as 0 (0%), 1 (1%–25%), 2 (26%–50%), 3 (51%–75%), 4 (76%–100%).

### 4.7 Identification of RREB1‐interacting proteins by LC–MS/MS

HCT116 cells stably overexpressing RREB1 cultured in 10 cm dishes were treated with or without 50 µM 5‐FU for 24 h, and then lysed with Co‐IP buffer. Co‐IP was conducted by adding 20 µL anti‐Flag beads (HY‐K0207‐1, MCE) for overnight at 4°C. The co‐immunoprecipitated proteins were digested into peptides with trypsin for liquid chromatography with tandem mass spectrometry (LC–MS/MS) identification as methods we described before.^28^ In brief, data acquisition was performed on an easy nano‐LC1000 HPLC system (Thermo Scientific) and a Q‐Exactive mass spectrometry (Thermo Scientific). Raw data searching was processed in MaxQuant software (version 2.2) with a human uniprot database (date to 2023.04).

### 4.8 Cell cycle and apoptosis detection

For cell cycle assays, 1 × 10^5^ cells were seeded in 6‐well plate and FBS starvation was treated for 24 h, cell cycle was detected using a commercial kit (KGA511, KeyGen BioTECH). For apoptosis detection, HCT116 cells were seeded into 6‐well plate and treated with 5‐FU for 24 h. Then the cells were harvested and stained with Annexin V‐APC and 7‐AAD using an apoptosis detection kit (E‐CK‐A218, Elabscience) for flow cytometry analysis.

### 4.9 ChIP

The binding of RREB1 to the promoter region within target gene was detected using ChIP assay (#9003, Cell Signaling Technology). Cells were cultured with 5‐FU exposure for 24 h, and cells were fixed with final 1% formaldehyde. Fixed chromatin was then washed by cold PBS and lysed to obtain nuclei for a further digestion with micrococcal nuclease to 150–900 bp DNA fragment, followed by three sets of sonication with 20 s pulse and 20 s gap. IPs were conducted by adding 8 µg primary anti‐Flag M2 mouse antibody (F1804, Sigma‐Aldrich) to enrich chromatin for overnight incubation at 4°C, followed by adding 30 µL of protein G magnetic beads for 2 h incubation. Purified DNA was extracted with supported spin collections. Next, we proceed to perform standard PCR or qPCR analysis to quantify the binding of RREB1 to target genes with ChIP‐qPCR primers as follows; TK1‐Forward: TTAGTCCCTCCCTGCAATCC; TK1‐Reverse: GGTCCGCCCACCAAGTTTA; TS‐Forward: TGCAAATCCCTTATTA

GTTGTAGG; TS‐Reverse: CCCTTTGGGAACCGTCTGG.

### 4.10 Comet assay to detect DNA damage

DNA damage caused by 5‐FU in CRC cells was measured with an alkaline comet assay kit (4250‐050‐K, Trevigen). In brief, cells were seeded in slides with a low‐melt agarose and lysed with lysis solution. DNA is electrophoresis at 21 V for 30 min, followed by an ethanol fixation and propidium iodide staining. The presence of comet tail was examined with fluorescence microscope. Tail moment was calculated by CASP software.

### 4.11 Subcutaneous xenograft model in nude mice

The animal experiments were performed in accordance with the guidelines of the Institutional Animal Care and Use Committee (Approval number: 20220531054). All efforts were made to minimize animal suffering and to reduce the number of animals used.

A total of 5 × 10^6^ HCT116 cells stably expressing Flag‐RREB1 or Flag‐RREB1^3KR^ as well as Flag‐Control were subcutaneously injected into 4‐week‐old BALB/c nude mice. Tumor volumes were recorded every 3 days. Tumor volumes were measured using a digital caliper and calculated using the formula: volume = 0.5 × length × width^2^. After 21 days, mice were euthanized, and tumors were excised and weighed. Tumors were then snap‐frozen in liquid nitrogen or fixed in 4% paraformaldehyde for further protein analysis and histological analysis.

### 4.12 Online public database

We downloaded public datasets from TCGA (https://portal.gdc.cancer.gov/) and GEO database (https://www.ncbi.nlm.nih.gov/geo/) including GSE25070, GSE44861, GSE44076, GSE39582 and GSE38832 to evaluate the expression of RREB1 in CRC.

For SUMOylation site prediction software, we analyzed putative RREB1 SUMOylation sites in online websites (SUMOplot, http://www.abgent.com/sumoplot; GPS‐SUMO, http://sumosp.biocuckoo.org/). For RREB1 binding sequence prediction within target gene promoter, we downloaded promoter sequence of target genes from NCBI, and then promoter sequence was analyzed in online website (http://jaspar.genereg.net/).

### 4.13 Statistical analysis

Statistical analysis was performed using GraphPad Prism 7. Data were represented as mean ± SD (standard deviation) from three repeats. *p*‐value <0.05 was considered as a statistically significant difference. Differences between two groups were analyzed by two‐tailed unpaired Student's *t*‐test or paired Student's *t*‐test. Two‐way analysis of variance (ANOVA) was used to analyze the statistical of cell cycle distribution.

## AUTHOR CONTRIBUTIONS

Ya‐nan Deng: Methodology, Investigation, Data analysis, Writing – original draft, Writing – review & editing, Visualization. Lan Huang, Shan Gao, and Zenghua Sheng: Investigation. Nan Zhang: Investigation, Visualization. Yinheng Luo: Data analysis, Visualization. Samina Ejaz Syed, Ruiwu Dai, Qiu Li, and Xianghui Fu: Resources. Shufang Liang: Methodology, Resources, Writing – original draft, Writing – review & editing, Supervision, Project administration, Funding acquisition. All the authors read and approved the final manuscript.

## CONFLICT OF INTEREST STATEMENT

The authors declare no conflicts of interest.

## ETHICS STATEMENT

The animal experiments were performed in accordance with the guidelines of Sichuan University and approved by the Animal Care Committee of Sichuan University (Approval number: 20220531054). The CRC clinical samples were collected from General Hospital of Western Theater Command, which was approved by the Ethics Committee of Western Theater General Hospital (approval number: 2024EC3‐ky004). Informed consent was obtained from all participants in the study.

## Supporting information



Supporting Information

## Data Availability

The datasets generated during and/or analyzed in this study are available from the main text and Supporting Information file, as well as corresponding author SFL. RNAseq raw data are available in Science Data Bank (https://cstr.cn/31253.11.sciencedb.16219).
