# Supplementary material for: The SUMOylated RREB1 interacts with KDM1A to induce 5‐fluorouracil resistance via upregulating thymidylate synthase and activating DNA damage response pathway in colorectal cancer
Source: MedComm (2020). 2025 Feb 21;6(3):e70105. doi: 10.1002/mco2.70105 (PMC11843160; doi:10.1002/mco2.70105)
Supplement: Supplementary file 1 — Supporting Information [file MCO2-6-e70105-s001.pdf]

Supplementary information

The SUMOylated RREB1 interacts with KDM1A to induce 5-fluorouracil resistance via upregulating thymidylate synthase and activating DNA damage response pathway in colorectal cancer

Running title: RREB1 contributes to 5-FU resistance in colorectal cancer

Ya-nan Deng[1], Lan Huang[1], Shan Gao[1], Zenghua Sheng[1], Yinheng Luo[1], Nan Zhang[2], Samina Ejaz Syed[3], Ruiwu Dai[4], Qiu Li[2], Xianghui Fu[1], Shufang Liang[1*]

1 Department of Biotherapy， Cancer Center and State Key Laboratory of Biotherapy, West China Hospital, Sichuan University, Chengdu, P.R. China.

2 Department of Medical Oncology, Cancer Center West China Hospital, Sichuan University, Chengdu, P.R. China.

3 Department of Biochemistry and Biotechnology, Baghdad Campus, The Islamia University of Bahawalpur, Pakistan

4 Department of General Surgery, General Hospital of Western Theater Command, Chengdu, P.R. China.

* To whom correspondence should be addressed: Dr. Shufang Liang, Department of Biotherapy, Cancer Center and State Key Laboratory of Biotherapy, West China Hospital, Sichuan University, No.17, Section 3 of Renmin South Road, Chengdu 610041, P.R. China. E-mail: zizi2006@scu.edu.cn

Figure S1

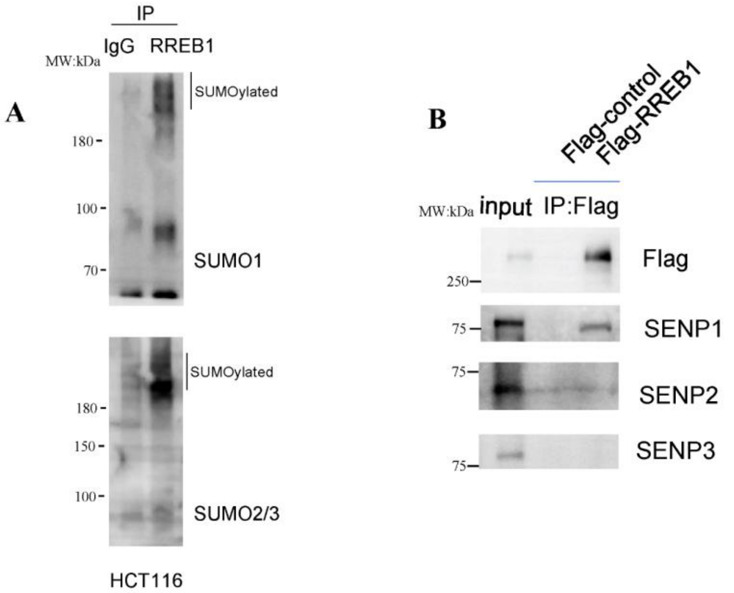

Figure S1. Cell endogenous RREB1 is a substrate of SUMOylation. (A) Cell endogenous RREB1 can be SUMOylated by SUMO1 and SUMO2/3, which was detected by using antibody against RREB1 (Proteintech, 20280-1-AP) in HCT116 cells. (B) RREB1 has no interaction with SENP2 and SENP3. The pFlag-RREB1 plasmids were transfected into HCT116 cells for 48h, and Co-IP was performed by anti-Flag M2 antibody as indicated. Then, SENP2 and SENP3 was detected by western blot assay.

Figure S2

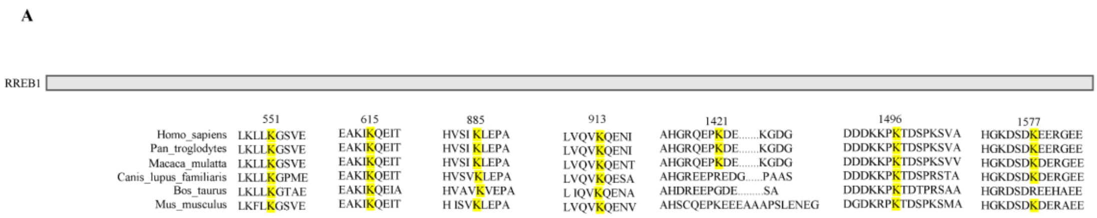

Figure S2. Sequence alignment analysis of lysine at 551, 615, 885, 913, 1421, 1496 and 1577 in RREB1 among various species. Upper lane is RREB1 gene model. Yellow marks the lysine at each site.

Figure S3

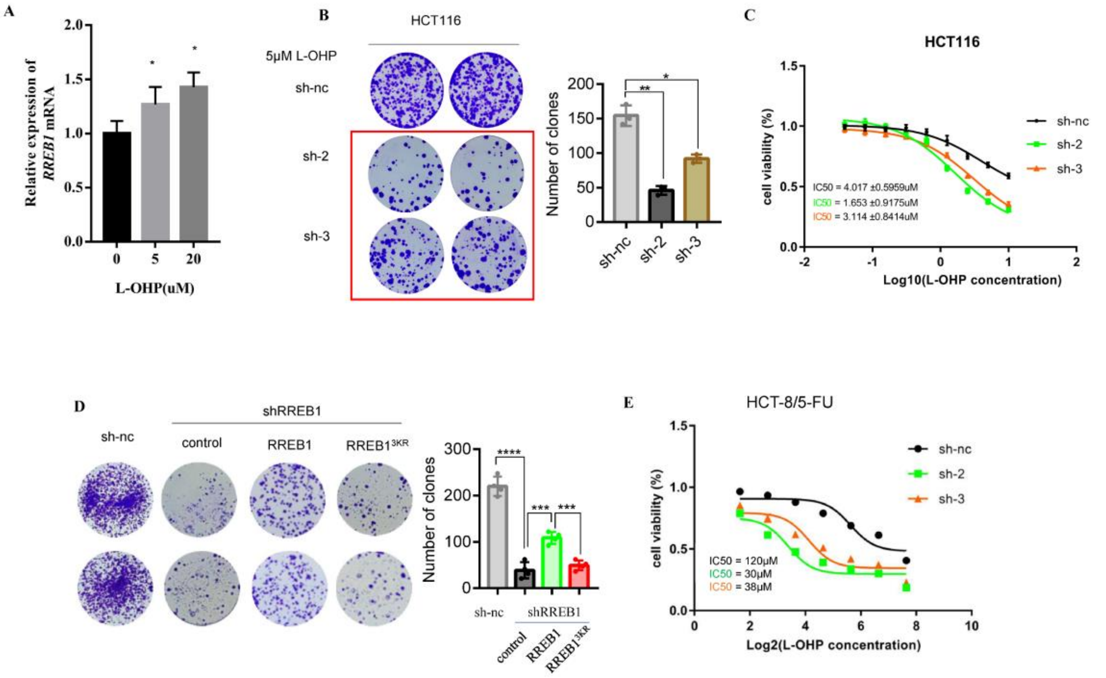

Figure S3. Colony formation and IC_{50} detection in RREB1 knockdown cells in response to oxaliplatin treatment. (**A**) *RREB1* mRNA detection by qPCR in HCT116 cells treated with 0μM, 5μM and 20μM oxaliplatin for 24h. *β-actin* was used as an internal control. (**B**) Colony formation assay under 5μM oxaliplatin treatment for 12 days in HCT116 cells with RREB1-knockdown (sh-2 and sh-3) or with negative control (sh-nc). The statistic by the right side shows that the clone number in sh-2 and sh-3 was significantly reduced compared with sh-nc. (**C**) The cell viability assay shows that the RREB1-knockdown (sh-2 and sh-3) in HCT116 cells decreased the IC_{50} of oxaliplatin. (**D**) plasmids of pControl, pRREB1, $pRREB1^{3KR}$ were transfected into shRREB1 HCT116 cells. Then 2500 cells were seeded into 6-well plate for 12d, DMEM supplemented with 1μM 5-FU. After fixation, cells were stained and counted. (**E**) The cell viability assay shows that the RREB1-knockdown (sh-2 and sh-3) in HCT-8/5-FU cells decreased the IC_{50} of oxaliplatin. *p<0.05, **p<0.01, ***p=0.0001

Figure S4

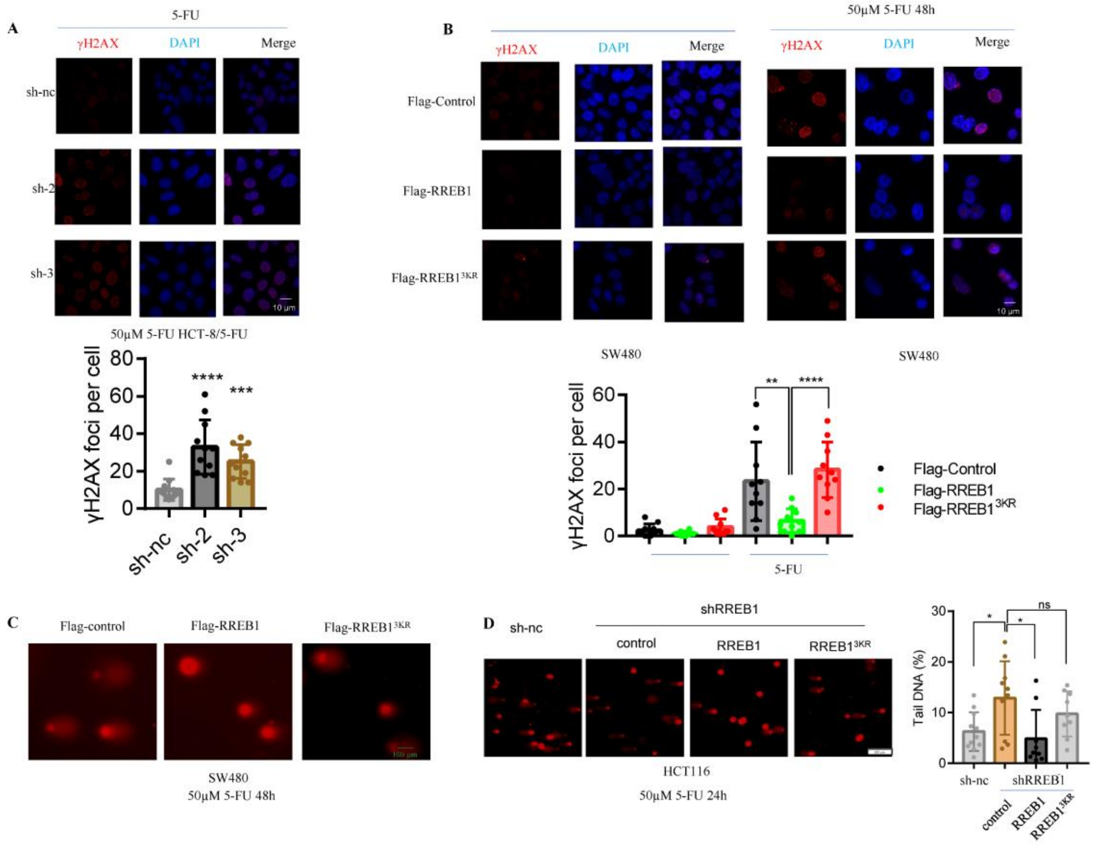

Figure S4. γH2AX foci and DNA damage analysis in HCT-8/5-FU or SW480 cells(**A**) 5-FU-induced DNA damage, marked by γH2AX, was evaluated by immunofluorescence in RREB1-knockdown HCT-8/5-FU cells (sh-2 and sh-3). A final concentration at 50µM of 5-FU was added into RPMI-1640 for 48h. Then cells were fixed and stained by γH2AX antibody. (**B**) 5-FU-induced DNA damage, marked by γH2AX, was evaluated by immunofluorescence in Flag-RREB1- or Flag-RREB1^{3KR}-overexpression SW480 cells. Cells were treated with or without 50µM 5-FU for 48h. (**C**) Comet assay was conducted to assess DNA damage in Flag-RREB1- or Flag-RREB1^{3KR}-overexpression SW480 cells after 50µM 5-FU treatment for 48h. (**D**) 50µM 5-FU incubation was treated in sh-nc HCT116 cells, and RREB1 knock-down HCT116 cells with overexpressing RREB1 or RREB1^{3KR}. A subsequent comet assay was conducted to detect the effect of RREB1 and RREB1^{3KR} on the DNA protection. Scale bars: 100µm. *p<0.05, **p<0.01, ****p<0.0001

Figure S5

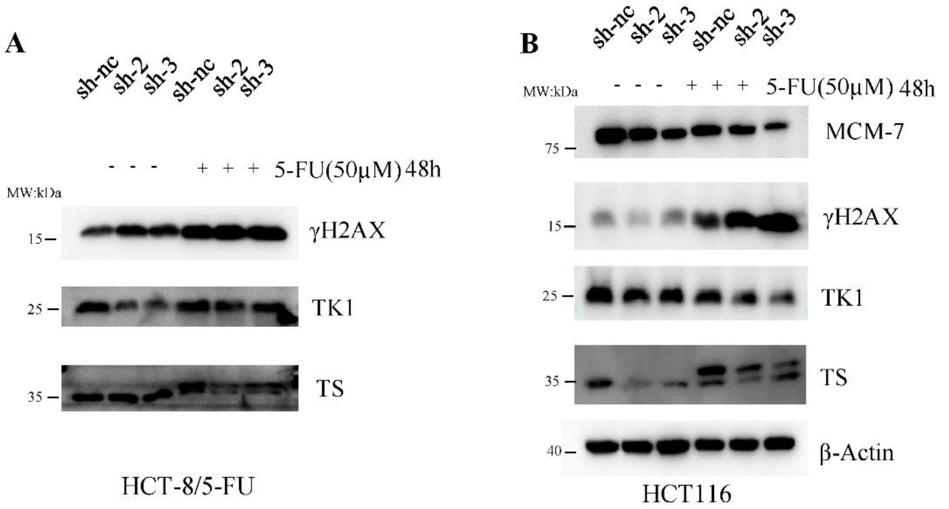

Figure S5. Western blot analysis of TS and TK1 in RREB1 knockdown CRC cells (**A&B**) RREB1-knockdown by sh-2 and sh-3 in HCT-8/5-FU cells (**A**) and HCT116 cells (**B**) were treated with or without 50μM 5-FU for 48h. Then cell lysates were collected for western blot analysis on TS, TK1, γH2AX and MCM-7. β-Actin was used as an internal control.

Figure S6

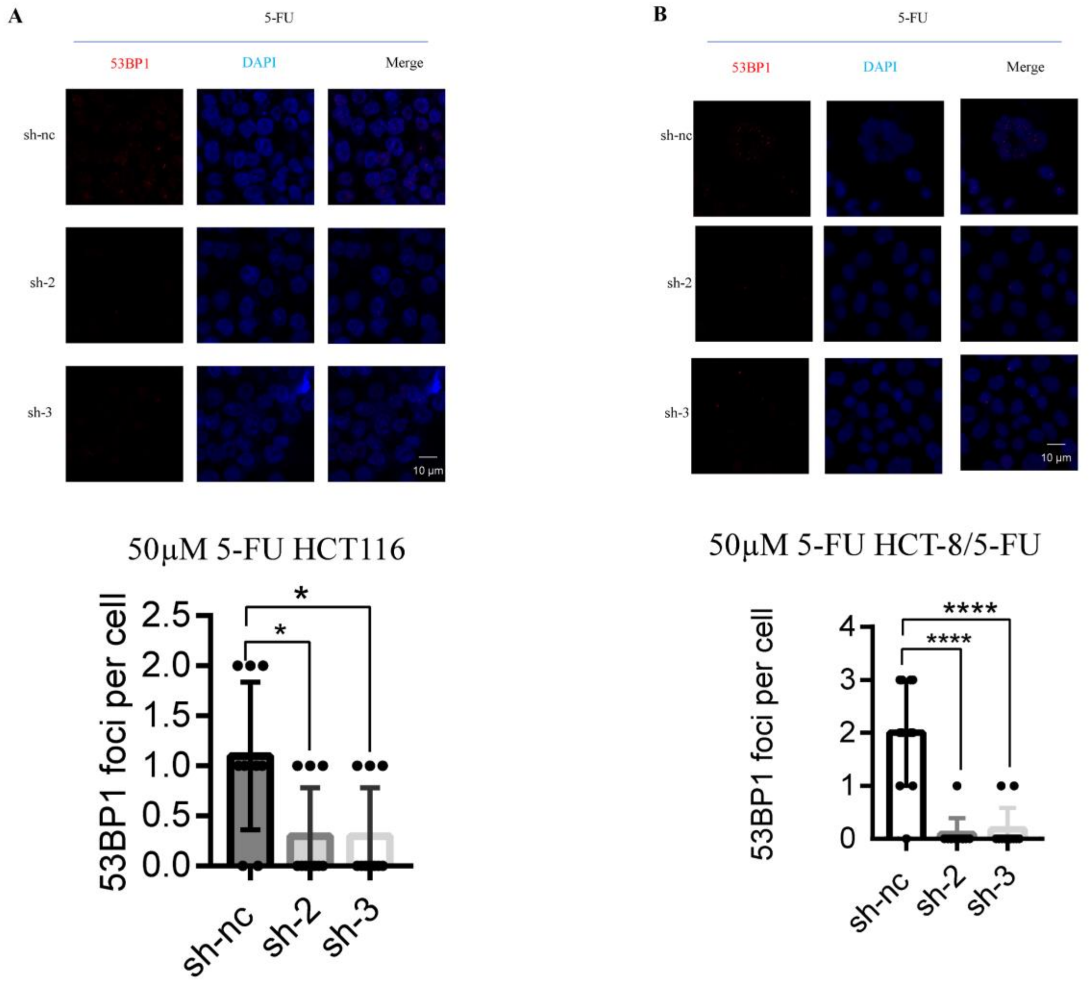

Figure S6. 53BP1 foci formation analysis in RREB1 knockdown cells (**A&B**) Immunofluorescence assay to evaluate the effect of RREB1 on the recruitment of 53BP1 in RREB1-knockdown HCT116 cells (**A**) and HCT-8/5-FU cells (**B**) under treatment of 50μM 5-FU for 24h. *$p<0.05$, ****$p<0.0001$

Figure S7

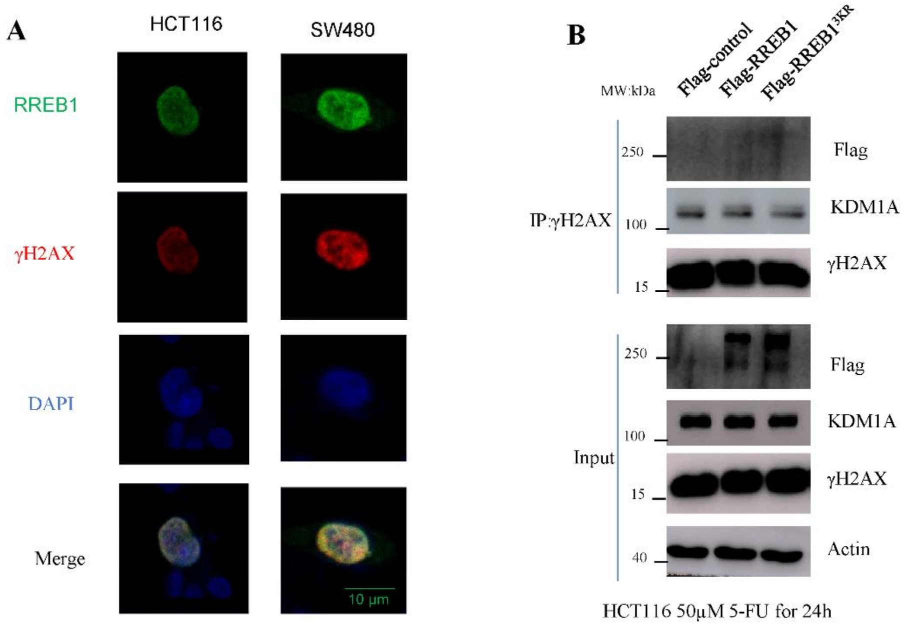

Figure S7. RREB1 interacts with γH2AX. (**A**) Co-localization analysis between RREB1 and γH2AX. Flag tagged RREB1 was transfected into HCT116 and SW480 cells, and then cells were treated with 50µM 5-FU for 24h. After cells fixation, γH2AX and RREB1 were stained with anti-γH2AX and anti-Flag antibody for immunofluorescence. (**B**) γH2AX interacts with RREB1 under 5-FU treatment. Flag-RREB1-, Flag-RREB13KR- and Flag-Control-HCT116 were treated with 50µM 5-FU for 24h, and then cell lysates were collected for Co-IP using anti-γH2AX antibody. Elutes were detected by western blot with anti-Flag, anti-KDM1A and anti-γH2AX antibody.

Figure S8

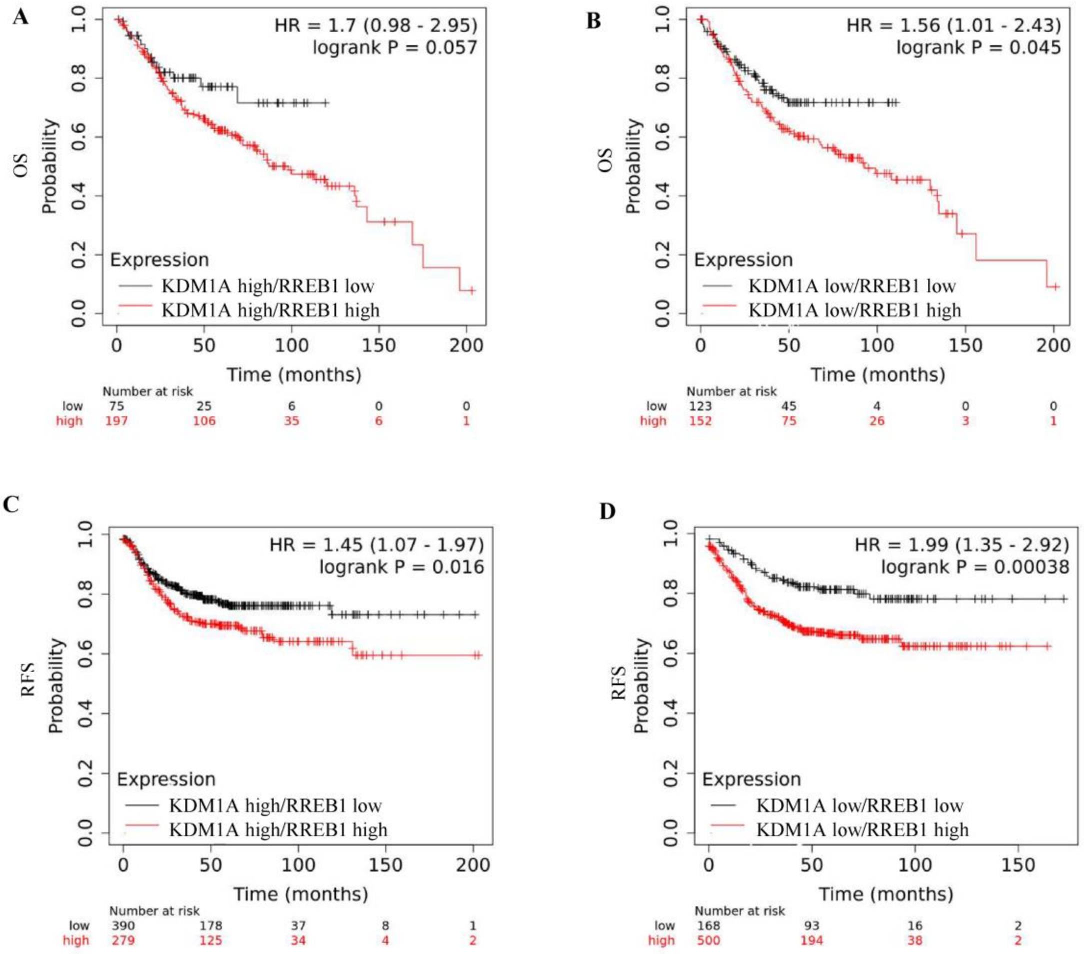

Figure S8. Combination of KDM1A and RREB1 for overall survival (OS) and relapse-free survival (RFS) analysis in an online website (www.kmplot.com/) based on Colon cancer datasets (GSE12945, GSE13294, GSE14333, GSE143985, GSE17538, GSE18088, GSE26682, GSE30540, GSE31595, GSE33114, GSE34489, GSE37892, GSE38832, GSE39582, GSE41258, GSE92921). (**A**) Patients with KDM1A high expression were divided into two groups based on median RREB1 mRNA expression for OS analysis. (**B**) Patients with KDM1A low expression were divided into two groups based on median RREB1 mRNA expression for OS analysis. (**C**) Patients with KDM1A high expression were divided into two groups based on median RREB1 mRNA expression for RFS analysis. (**D**) Patients with KDM1A low expression were divided into two groups based on median RREB1 mRNA expression for RFS analysis.

Figure S9

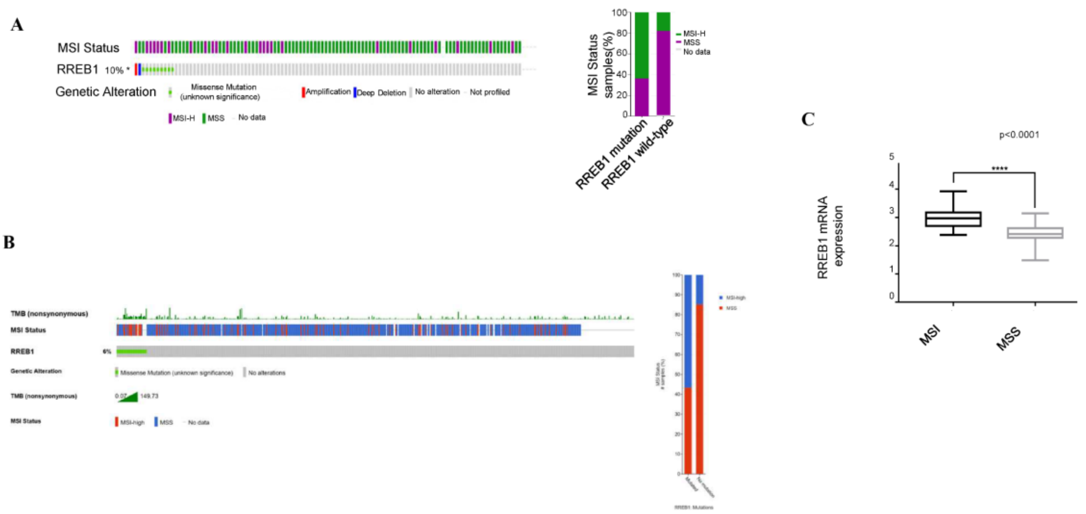

Figure S9. RREB1 shows an association with microsatellite instability (**A&B**) RREB1 variations were associated with the microsatellite instability high (MSI-H) status. Upper lane shows the MSI status of each colorectal cancer patient, and lower lane shows the RREB1 status. MSS: microsatellite stable. No data: the status of MSI in this sample was not available. Analysis was performed in cBioPortal online website (https://www.cbioportal.org). The statistic of MSI status in patients with RREB1 mutation and RREB1 wild-type was shown on the right side. The MSI-H was significantly enriched in RREB1 mutation group compared with RREB1 wild-type group. (**C**) The relative expression of RREB1 mRNA in colorectal cancer patients with microsatellite instability (MSI) and microsatellite stable (MSS), data from TCGA database. ****p<0.0001

Figure S10

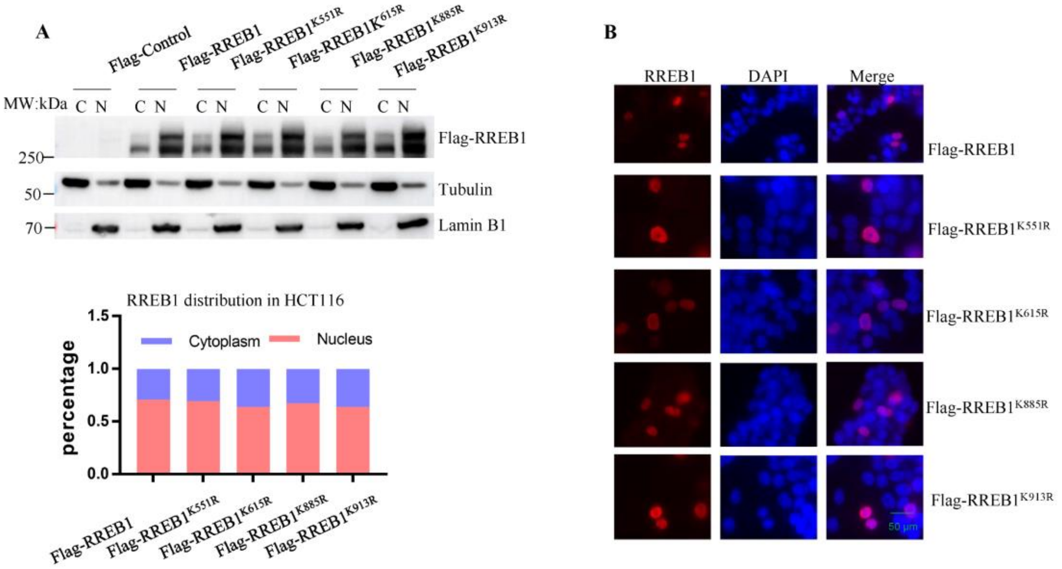

Figure S10. SUMOylation shows no effect on RREB1 localization. (**A**) Flag-RREB1 and Flag-RREB1 mutants at lysine site 551, 615, 885 and 913 were transfected in HCT116 cells. Then, cytoplasm (C) and Nucleus (C) were isolated using Nuclear and Cytoplasmic Protein Extraction Kit (Beyotime, P0027) for western blot detection. (**B**) Immunofluorescence assay was applied to evaluate the localization of RREB1 and RREB1 mutants.

Table S1. SUMOylation site on RREB1 predicted by online tools

gps-SUMO				SUMO-plot		
site		score		site		Score
203	QSGDLEKKADEVFHC	4.683		K885	PPHVS **IKLE** PASSF	0.94
615	LSMEAKIKQEITEGE	10.45		K615	SMEAK **IKQE** ITEGE	0.94
885	PPPHVSIKLEPASSF	23.24		K913	LALVQ **VKQE** NISFL	0.93
913	GLALVQVKQENISFL	28.984		K41	GSPQG **IKSP** SKPPG	0.84
945	SIPKNFRKGDKDLAT	4.513		K957	ATPSE **AKKP** EEEAG	0.69
1421	AHGRQEPKDEKGDGA	11.328		K1496	DDDKK **PKTD** SPKSV	0.61
1424	RQEPKDEKGDGASTA	4.499		K1421	HGRQE **PKDE** KGDGA	0.61
1444	PAPEQEEKPPETPAE	3.908		K624	ITEGE **LKAF** MTAPG	0.56
1496	SDDDKKPKTDSPKSV	5.661		K1577	HGKDS **DKEE** RGEED	0.5
1577	HHGKDSDKEERGEED	4.068		K1488	EKRSS **EKSD** DDKKP	0.5

Table S2. qPCR primers

qPCR-RREB1-F	CCATCTCCTCTGAAACGTAGGC
qPCR-RREB1-R	ACTCCTTGAAACATACTGGGCA
qPCR-β-actin-F	GGACCTGACTGACTACCTCAT
qPCR-β-actin-R	CGTAGCACAGCTTCTCCTTAAT
TK1-qPCR-F	GGGCAGATCCAGGTGATTCTC
TK1-qPCR-R	TGTAGCGAGTGTCTTTGGCATA
TYMS-qPCR-F	CTGCTGACAACCAAACGTGTG
TYMS-qPCR-R	GCATCCCAGATTTTCACTCCCTT
POLA2-QPCR-F	GAAGCGAGCTATCTCTACCCC
POLA2-QPCR-R	CCACTTCTCCTCGGTTACTTCG
MCM5-QPCR-F	GGAAGTGCAACACAGATCAGG
MCM5-QPCR-R	AGGGACGACCTTGTCACACA
MCM7-QPCR-F	ACTCTCAGAAACCTACCTGGAAG
MCM7-QPCR-R	CAGCTTTTCGTAGAAATCCTCCT
